# The trimer interface in the quaternary structure of the bifunctional prokaryotic FAD synthetase from *Corynebacterium ammoniagenes*

**DOI:** 10.1038/s41598-017-00402-6

**Published:** 2017-03-24

**Authors:** Ana Serrano, María Sebastián, Sonia Arilla-Luna, Silvia Baquedano, Beatriz Herguedas, Adrián Velázquez-Campoy, Marta Martínez-Júlvez, Milagros Medina

**Affiliations:** 1 Departamento de Bioquímica y Biología Molecular y Celular, Facultad de Ciencias, and Institute of Biocomputation and Physics of Complex Systems (Joint Units BIFI-IQFR and CBsC-CSIC), Universidad de Zaragoza, Zaragoza, Spain; 2 Fundación ARAID, Diputación General de Aragón, Zaragoza, Spain; 3 Aragon Institute for Health Research (IIS Aragon), Zaragoza, Spain; 4 Centro de Investigaciones Biológicas, CSIC, Ramiro de Maeztu 9, E-28040 Madrid, Spain; 5 MRC Laboratory of Molecular Biology, Francis Crick Avenue, CB2 0QH Cambridge, UK

## Abstract

Bifunctional FAD synthetases (FADSs) fold in two independent modules; The C-terminal riboflavin kinase (RFK) catalyzes the RFK activity, while the N-terminal FMN-adenylyltransferase (FMNAT) exhibits the FMNAT activity. The search for macromolecular interfaces in the *Corynebacterium ammoniagenes* FADS (*Ca*FADS) crystal structure predicts a dimer of trimers organization. Within each trimer, a head-to-tail arrangement causes the RFK and FMNAT catalytic sites of the two neighboring protomers to approach, in agreement with active site residues of one module influencing the activity at the other. We analyze the relevance of the *Ca*FADS head-to-tail macromolecular interfaces to stabilization of assemblies, catalysis and ligand binding. With this aim, we evaluate the effect of point mutations in loop L1c-FlapI, loop L6c, and helix α1c of the RFK module (positions K202, E203, F206, D298, V300, E301 and L304), regions at the macromolecular interface between two protomers within the trimer. Although none of the studied residues is critical in the formation and dissociation of assemblies, residues at L1c-FlapI and helix α1c particularly modulate quaternary architecture, as well as ligand binding and kinetic parameters involved with RFK and FMNAT activities. These data support the influence of transient oligomeric structures on substrate accommodation and catalysis at both *Ca*FADS active sites.

## Introduction

**Figure 1:**
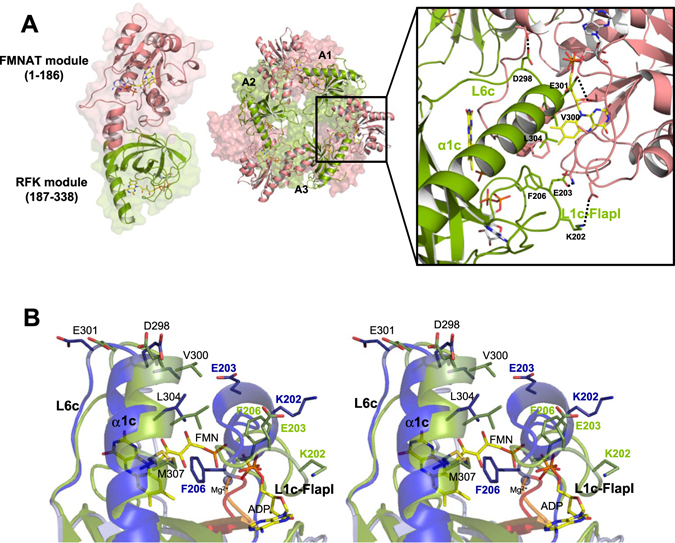
Structure of *Ca*FADS. (**A**) Cartoon representation of the *Ca*FADS monomer (PDB code: 2x0k) and of the dimer of trimers model predicted by the PISA server (one of the trimers is represented as a surface). The RFK module and the FMNAT module are colored in green and pink, respectively. The right panel shows a detail of the head-to-tail disposition between the RFK and FMNAT modules of neighboring protomers, within each one of the trimers. Residues involved in the stabilization of the trimer are shown as sticks and the ones mutated in the present work are labelled. H-bonds and salt-bridges established by these residues are indicated with dotted lines. Predicted positions for flavin and adenine nucleotide ligands are shown as sticks with carbons in yellow and gray, respectively. (**B**) Cross-eye stereo view of the superposition of the RFK modules of *Ca*FADS (PDB code: 2x0k; green) and the ternary complex *Ca*FADS RFK:FMN:ADP:Mg^2+^ (PDB code: 5a89; blue). Relevant residues are shown in sticks. FMN, ADP (in sticks CPK colored with C in yellow) and Mg^2+^ (blue sphere) are from the ternary complex structure. Backbones of the PTAN motifs (207–210 residues) of *Ca*FADS and the ternary complex are highlighted in orange and red, respectively.

Many biological events require the formation of protein oligomers or multiprotein assemblies, rendering this formation a mechanism for the modulation of protein activity^1–3^. Protein-protein association or dissociation processes can be regulated by the binding of partner proteins, metal cofactors or small allosteric effectors, as well as the conformational changes coupled to catalysis^4, 5^. The FAD synthetase from *Corynebacterium ammoniagenes* (*Ca*FADS) is a bifunctional enzyme responsible for the synthesis of flavin mononucleotide (FMN) and flavin adenine dinucleotide (FAD) cofactors. The *Ca*FADS crystal structure shows that this protein folds in two nearly independent modules, similar to other bifunctional prokaryotic FADSs^6, 7^. The C-terminal module catalyzes FMN synthesis from riboflavin (RF) (riboflavin kinase, RFK), while the N-terminal one transforms FMN into FAD (FMN:adenylyltransferase, FMNAT) (Figure 1A). Bioinformatic tools for the exploration of macromolecular interfaces predict that *Ca*FADS might stabilize in hexameric assemblies in solution, organized as dimer of trimers^6, 8^. Some experimental facts support the formation of these assemblies: 1) the identification in cultures of *C. ammoniagenes* cells of different oligomeric forms of FADS and, 2) the observation at the single-molecule level of the dimer of trimers as well as of oligomers induced by ligand binding^8^. Therefore, it was proposed that binding and dissociation of substrates/products of the *Ca*FADS enzymatic activities, FMNAT and particularly RFK, induce conformational changes which lead to self-association/dissociation processes. No dependence on the protein concentration for these processes has been observed *in vitro* ^8, 9^.

In the predicted *Ca*FADS dimer of trimers model, the protomers adopt a head-to-tail organization within each trimer that causes the RFK and FMNAT catalytic sites of two neighboring protomers (Figure 1A)^6^ to be in proximity. Thus, in the dimer of trimers model, ligand binding and catalytic efficiency during catalysis at the active sites of one protomer might be modulated by the active sites of neighboring protomers. Structural analysis of the *Ca*FADS dimer of trimers suggested loop L3c (residues 231–246, c denotes for the C-terminal RFK module) is a determinant to stabilize this assembly^8^. It is unknown whether other prokaryotic FADSs might produce such macromolecular assemblies, but L3c is only predicted in FADSs from the taxonomically closely related *Corynebacterium* and *Mycobacterium* genera^10, 11^. Attempts to produce stable recombinant FADS from the *Mycobacterium* genus have so far failed, but the FADS from the human pathogen *M. tuberculosis* shares 45% identity with *Ca*FADS. Therefore, the latter enzyme is considered a good model for the former, as with other *M. tuberculosis* proteins^12^. Stabilization of a dimer of trimers might only occur in FADSs from these two genera, but whether any other family members could stabilize another type of assembly is still unknown. In this context, the formation of specific macromolecular assemblies, together with substrate inhibition or the requirement for reduced flavin substrates^13–16^, might be other regulatory strategies adopted by FADSs. These regulatory mechanisms are highly relevant because they regulate cellular processes such as flavin delivery to apo-flavoproteins or those dependent on flavin homeostasis^17, 18^.

In addition to loop L3c, several elements stabilize head-to-tail interfaces within each trimer through H-bonds, electrostatic and hydrophobic interactions in the *Ca*FADS dimer of trimers. Loop L4n (n denotes for N-terminal FMNAT module), loops L1c-FlapI and L6c, together with helix α1c, form the interface between two protomers within the trimer and contribute to the structures of the active sites (Figure 1A)^6^. L1c-FlapI, L6c and α1c, together with loop L4c-FlapII, form the closed conformation of the RFK flavin-binding site that is reached after being triggered by substrate/product binding (Figure 1B)^9^. In addition, a salt-bridge between the catalytic base at the RFK module, E268, and R66 at loop L4n stabilizes each trimer within the dimer of trimers^16, 19^, while residues at the RFK catalytic site modulate binding parameters and catalytic efficiency at the FMNAT active site^16^.

Here, at the molecular level, we analyze the relevance of the trimer head-to-tail interfaces in the stabilization of the quaternary assembly, as well as in ligand binding and catalysis. With this aim, we evaluate the effects produced by point mutations at L1c-FlapI (K202, E203 and F206), L6c (D298), and α1c (V300, E301 and L304) of *Ca*FADS (Figure 1)^6^. These secondary structural elements belong to the RFK module and contribute to the structure of the active sites for FMN and FAD synthesis of two contiguous protomers within each trimer (Figure 1A)^6^. Additionally, they are involved in important conformational changes related to flavin binding and catalysis at the RFK module (Figure 1B)^9^. Our data indicate that none of the mutated residues is critical for catalysis. Nevertheless, evaluation of the properties of the mutants, in view of the available 3D structures for wild-type (WT) and *Ca*FADS variants, indicates that the side chains replaced in this study contribute to stabilizing the oligomers, as well as the ligand binding and kinetic parameters for both enzymatic activities. Our results thus support the formation of organized transient oligomeric assemblies in the catalytic cycles of *Ca*FADS, and indicate that they contribute to modulating the *Ca*FADS efficiency during catalysis.

## Results

### Spectral properties of *Ca*FADS variants

The expression levels of all variants are similar to that of WT *Ca*FADS (10–17 mg per g cells). Most mutants primarily purified as monomers, with a small population of oligomeric species (Figure SP1), similar to WT *Ca*FADS^8, 19^. Far-ultraviolet (UV) circular dichroism (CD) spectra were similar to those of the WT^20^, indicating minor impact of the mutations on the enzyme’s secondary structure (Figures SP2A and SP2B). However, some mutations considerably decreased the intensity of the near-UV CD signal (Figures SP2C and SP2D), suggesting local changes in the region contributing to the signal.

Visible difference spectra monitored during titration with flavins indicated that all the *Ca*FADS variants interact with RF, FMN and FAD (Figure SP3). Noticeably, while mutations at charged residues increase the magnitude of the difference spectra in comparison to the WT enzyme, the opposite effect was observed for mutations at hydrophobic residues. The most significant changes of peak shapes in the spectra were observed when titrating with FAD and FMN, particularly for the F206W, D298E and E301K variants (Figures SP3C–SP3F), suggesting different environments of the isoalloxazine ring in the flavin binding site at the FMNAT module. The increase in the magnitude of the difference spectra upon titration of the preformed WT *Ca*FADS:ADP:Mg^2+^ complex with FMN (~8-times higher than in the absence of ADP) is related to the formation of the FMN binding site at the RFK module^16, 19, 20^. Noticeably, this effect was smaller for most of our variants and undetectable for E301K (Figures SP3G and SP3H). Calorimetric results (discussed below) indicate that, as reported for the WT, all variants maintain two binding sites for FMN in the presence of ADP. Difference spectra suggest that the mutations alter the closed conformation of the flavin isoalloxazine binding site at the RFK module, as described for WT^9^.

### Size distribution of *Ca*FADS variants

**Figure 2:**
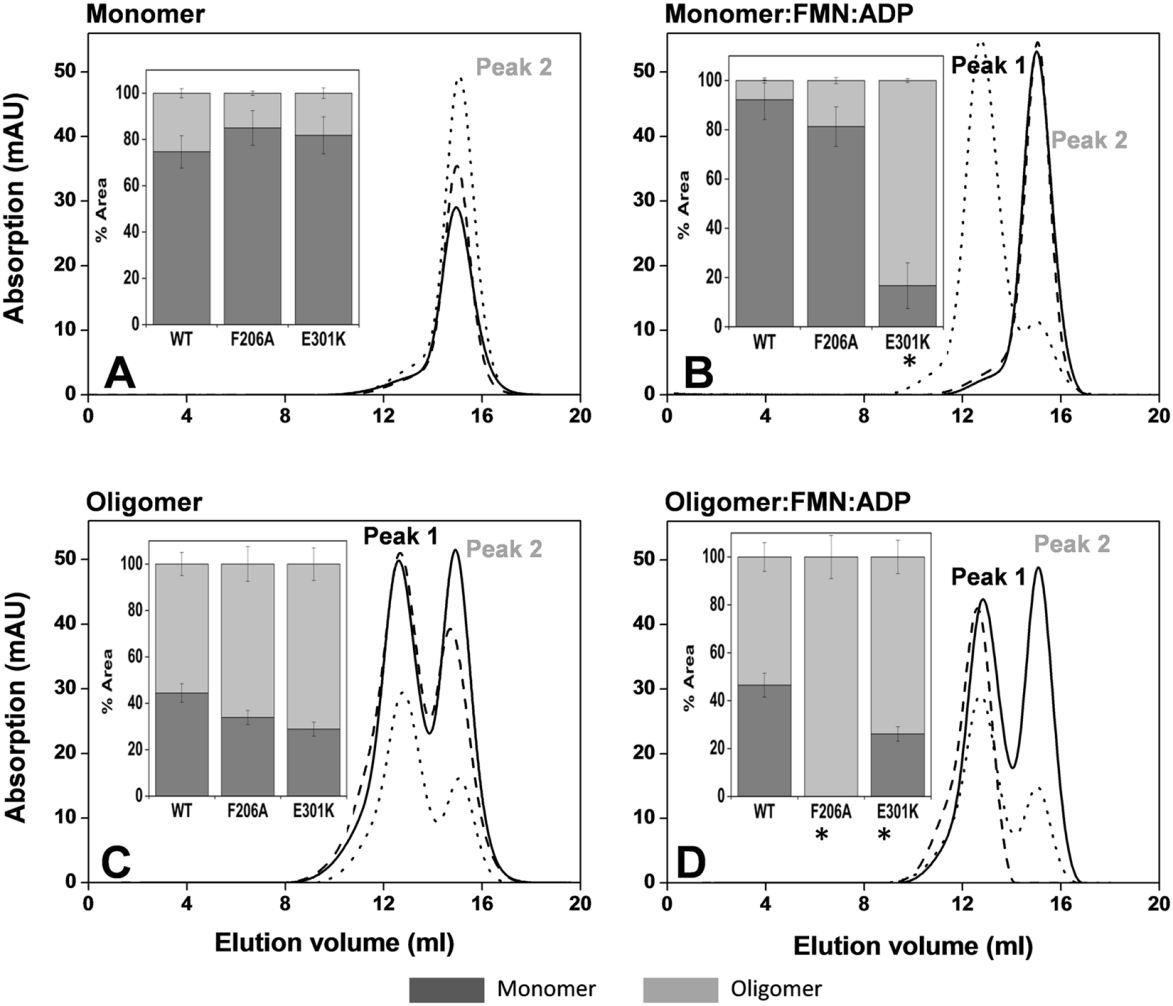
Gel filtration elution profiles of monomeric and oligomeric samples of WT (solid line), F206A (dashed line) and E301K (dotted line) *Ca*FADSs after incubation under different conditions. (**A**) The peak corresponding to the monomeric form, which was used as a control. (**B**) The peak corresponding to the monomeric form, after incubation with 25 μM FMN and 200 μM ADP. (**C**) The band corresponding to the oligomeric forms, which was also used as a control. (D) The band corresponding to the oligomeric forms, after incubation with 25 μM FMN and 200 μM ADP. The insets show the percentage of monomer (represented by the peak labelled peak 2) and the bulk of oligomeric species (labelled as peak 1) obtained from the chromatograms, with *indicating values that show statistically significant differences from the WT, as determined by the one-way ANOVA test (*P* < 0.002; n = 3, confidence interval 95%). All samples contained 15–20 µM of protein, 20 mM PIPES, 0.8 mM MgCl_2_, pH 7.0, and were incubated 10 min at room temperature before passing through a Superdex™ 200 10/300 GL column in the same buffer.

Previous studies showed that monomeric WT *Ca*FADS, incubated with the products of the RFK activity (FMN and ADP in the presence of Mg^2+^), stabilizes a transient dimer of trimers, which mostly dissociates into the monomer form upon the ligands’ removal by gel filtration chromatography^8, 19^. This is due to the protomers of *Ca*FADS rapidly assembling in response to ligand binding, and disassembling when ligands are eliminated. However, a small fraction of oligomers always remains after gel filtration, which probably reflects the presence of poorly fit assemblies with a large kinetic barrier to dissociation. This might be a consequence of the thermodynamic complexity of the system (see text and data below), which forms other assemblies than the transient dimer of trimers^8, 19^. In practice, these observations allow the evaluation of whether the introduced mutations modulate assembly and disassembly profiles of WT *Ca*FADS^8^. Taking advantage of this, we next studied whether the mutations influence the *Ca*FADS monomer-oligomer interconversion associated to the binding and dissociation of ligands. We used freshly purified fractions containing either monomeric or oligomeric (which included all oligomeric fractions isolated by gel filtration of freshly purified variants) species and independently incubated them with the products of RFK activity. Monomeric and oligomeric species in the absence of ligands were similarly treated but used as controls. Gel filtration chromatography was then used to remove ligand and separate monomers and oligomers and quantify their relative percentages (Figures 2 and SP4). For all variants, in the absence of ligands, samples that began as freshly prepared monomers eluted again mostly as monomers (peak labelled 2 in Figures 2A, SP4A and SP4B). Variants, when prepared as monomers that had been incubated with the products of the RFK activity, were also isolated mainly as monomers after gel filtration (Figures 2B, SP4C and SP4D), as has been reported for WT *Ca*FADS^8, 19^. The only exception was E301K *Ca*FADS, which recovered less than 20% of the monomer.

When oligomeric fractions (the peak labelled 1 in the chromatograms) were similarly treated in the absence of ligands, the monomer/oligomer ratio was ~0.8 for WT, with no statistically relevant differences for most of the variants (Figures 2C, SP4E and SP4F). This agrees with the large amount of oligomeric assemblies that remain, which, as mentioned above, possess a large kinetic barrier to disassembly^8, 19^. Nevertheless, this ratio significantly increased for the F206W, V300K and L304 variants, indicating the greater conversion of such assemblies into monomers (panel to the right of Figures SP4E and SP4F). Finally, when the original oligomeric fractions were treated with FMN and ADP, most of the variants exhibit monomer/oligomer ratios similar to the corresponding control. However, no traces of monomer were detected for the F206A variant (Figure 2D), indicating that this mutation prevents recovery of misfit assemblies by ligand binding and dissociation. In contrast, the V300K and L304K mutations clearly promote the recovery of the monomeric form (Figures SP4E and SP4F).

Altogether, these data indicate that point mutations introduced at F206 in L1c-FlapI and V300, E301 and L304 in α1c modulate the reestablishment of monomeric forms as well as the type of assemblies that are produced.

### Effects of the mutations in the catalytic activities of *Ca*FADS variants

Qualitative analysis of RFK and FMNAT reactions by thin layer chromatography (TLC) indicates that all the variants retained both catalytic activities (not shown).

The RFK activity was quantitatively analyzed by monitoring the conversion of RF into FMN and FAD (because there is no way to fully avoid FMNAT activity) (Table 1). At saturating ATP concentrations, all the variants exhibit the WT-like RF-inhibition kinetic profile (not shown). The calculated inhibition constants suggest that only mutation L304A slightly reduces the RF inhibitory effect (see *K*_i_ in Table 1), while the effect is large enough for F206W, D298E, V300A and E301K variants to prevent determination of accurate kinetic parameters. All mutants except V300K and L304K reduce both *k*_cat_ (1.5–8 times lower than WT *Ca*FADS) and *K*_m_^RF^ values (up to 4-fold lower), maintaining the catalytic efficiency of RF transformation within a factor of three from that of WT *Ca*FADS (Table 1). When determining the RFK activity as a function of ATP concentration, (experiments performed at RF concentrations producing 80% of the maximal measured activity), most of the variants show a significant ^app^ *k*_cat_ decrease (Table 1), except for F206A, F206K, V300K, L304A and L304K. Under such conditions, the introduced mutations significantly increased *K*_m_^ATP^ in the cases of non-conservative mutations at F206 and E203A, and slightly decreased its value for V300K, L304A and L304K (4–7 times less). When RF is kept constant, most of the variants reduce the catalytic efficiency for ATP and only V300K and variants with mutations at L304 show higher efficiency, because of an stronger apparent affinity for ATP (Table 1). These observations indicate that although the mutated residues are not critical to maintain the RFK activity, they are in some way implicated in the adequate allocation of substrates during catalysis.

**Table 1:** Steady-state kinetic parameters for the RFK activity (RF + ATP → FMN + ADP) of the different *Ca*FADS variants (n = 3; means ± SE).

	*k*_cat_^a,b^ (min^−1^)	*K*_m_^RF a,b^ (µM)	*K*_*i*_^a,b^ (µM)	*k*_cat_/*K*_m_^RF a,b^ (min^−1^ µM^−1^)	*k*_cat_^c^ (min^−1^)	*K*_m_^ATP c^ (µM)	*k*_cat_/*K*_m_^ATP c^ (min^−1^ µM^−1^)
**WT**	408 ± 230	11.7 ± 3.0	4.9 ± 3.9	34.9 ± 21.6	155 ± 5	28.2 ± 3.9	5.5 ± 0.8
**K202A**	63.8 ± 11.7	0.8 ± 0.4^e^	19.4 ± 8.5	9.9 ± 5.3	34.5 ± 1.0^e^	52.7 ± 5.3	0.66 ± 0.07
**E203A**	128 ± 67	3.7 ± 1.3	3.5 ± 3.2	34.6 ± 21.8	50.1 ± 2.8^e^	89.2 ± 13.9^e^	0.56 ± 0.09
**F206A**	189 ± 70	3.2 ± 1.9	9.5 ± 5.0	59.7 ± 41.8	139 ± 12	121 ± 29^e^	1.14 ± 0.3
**F206K**	176 ± 25	4.0 ± 1.2	29.0 ± 8.3	44.4 ± 14.7	150 ± 8	140 ± 24^e^	1.07 ± 0.2
**F206W**	> 50^d^	n.d.^d^	n.d.^d^	n.d.^d^	70.2 ± 6.0^e^	24.7 ± 11.1	2.84 ± 1.3
**D298A**	78.4 ± 18.9	0.7 ± 0.4^e^	8.7 ± 3.9	9.1 ± 5.6	32.2 ± 0.8^e^	26.6 ± 2.8	1.2 ± 0.13
**D298E**	>20^d^	n.d.^d^	n.d.^d^	n.d.^d^	20.9 ± 0.8^e^	64.9 ± 6.9	0.32 ± 0.03
**V300A**	>50^d^	n.d.^d^	n.d.^d^	n.d.^d^	69.7 ± 1.4^e^	37.5 ± 2.5	1.86 ± 0.13
**V300K**	359 ± 145	9.7 ± 6.4	31.8 ± 24.1	36.8 ± 28.5	187 ± 10^e^	7.25 ± 2.9	25.8 ± 10.4
**E301A**	53.0 ± 15.9	0.1 ± 0.3^e^	18.0 ± 11.9	10.2 ± 30.7	36.7 ± 1.1^e^	30.9 ± 4.1	1.2 ± 0.2
**E301K**	>30^d^	n.d.^d^	n.d.^d^	n.d.^d^	59.9 ± 2.0^e^	59.3 ± 6.4	1.0 ± 0.1
**L304A**	260 ± 63	3.3 ± 1.9	58.4 ± 40.7 ^f^	78.8 ± 49.2	177 ± 10	4.0 ± 3.0	44.3 ± 33.3^f^
**L304K**	540 ± 201	14.9 ± 5.7	5.0 ± 2.8	36.4 ± 19.4	131 ± 6	4.8 ± 2.8	27.4 ± 16

Data obtained at 25 °C in 20 mM PIPES pH 7.0, 0.8 mM MgCl_2_.

^a^Determined at saturating ATP concentrations.

^b^Inhibition by substrate prevented the determination of true parameters and these correspond to apparent constants; ^app^ *k*_cat_ and ^app^ *K*_m_. Estimated errors in ^app^ *k*_cat_ and ^app^ *K*_m_ values can increase up to ± 35% for the larger *K*_i_ values.

^c^Parameters estimated using an RF concentration at which ~80% of maximal activity is exhibited.

^d^Despite these variants being active, the high degree of inhibition prevented mathematical determination of their kinetic parameters.

^e^Values showing statistically significant differences, *P* < 0.002, from the WT, as determined by the one-way ANOVA test (n = 3, confidence interval 95%).

^f^Values showing statistically significant differences, *P* < 0.02, from the WT, as determined by the one-way ANOVA test (n = 3, confidence interval 95%).

The effects of the mutations on FMNAT activity were similarly evaluated (Table 2). The mutations produced minor effects on the *k*_cat_ and *K*_m_^ATP^ values (generally within a factor of two of those of the WT), but decreases in the *K*_m_^FMN^ were, in general, more significant (up to 25-fold in the case of E301A). Thus, these variants were more efficient than WT in transforming FMN. These data suggest that mutated residues at the RFK module of *Ca*FADS modulate the catalytically competent binding of FMN during the FMNAT activity of the enzyme.

**Table 2:** Steady-state kinetic parameters for the FMNAT activity (FMN → FAD) of the different *Ca*FADS variants (n = 3; means ± SE).

	*k*_cat_ (min^−1^)	*K*_m_^FMN^ (µM)	*K*_m_^ATP^ (µM)	*k*_cat_/*K*_m_^FMN^ (min^−1^ µM^−1^)	*k*_cat_/*K*_m_^ATP^ (min^−1^ µM^−1^)
**WT**	5.5 ± 0.5	10.1 ± 1.0	22.4 ± 2.0	0.54 ± 0.07	0.25 ± 0.03
**K202A**	2.3 ± 0.2^a^	2.9 ± 0.5^a^	12.1 ± 2.9	0.80 ± 0.10	0.19 ± 0.05
**E203A**	3.2 ± 0.2^a^	0.70 ± 0.10^a^	10.8 ± 2.6	4.5 ± 0.7^a^	0.30 ± 0.07
**F206A**	4.2 ± 0.2^a^	2.9 ± 0.7^a^	38.0 ± 7.2	1.45 ± 0.4	0.11 ± 0.02
**F206K**	6.2 ± 0.3	5.4 ± 0.6^a^	38.8 ± 3.3	1.15 ± 0.1	0.16 ± 0.02
**F206W**	4.6 ± 0.2	1.7 ± 0.5^a^	25.2 ± 6.1	3.3 ± 1	0.22 ± 0.05
**D298A**	6.1 ± 0.4^a^	1.2 ± 0.2^a^	20.7 ± 6.4	5.1 ± 0.9^a^	0.30 ± 0.09
**D298E**	3.3 ± 0.3	0.95 ± 0.30^a^	10.4 ± 3.3	3.47 ± 1.1	0.31 ± 0.10
**V300A**	4.9 ± 0.2	1.4 ± 0.2^a^	46.2 ± 7.6^a^	3.5 ± 0.5	0.11 ± 0.02
**V300K**	4.6 ± 0.3	8.3 ± 1.7	9.5 ± 1.4	0.55 ± 0.10	0.48 ± 0.08
**E301A**	6.9 ± 0.2	0.42 ± 0.02^a^	19.7 ± 3.6	16.4 ± 0.9^a^	0.35 ± 0.06
**E301K**	6.3 ± 0.5	0.85 ± 0.20^a^	15.3 ± 3.6	7.4 ± 1.8^a^	0.41 ± 0.10
**L304A**	2.5 ± 0.2^a^	2.8 ± 0.3^a^	34.7 ± 9.4	0.91 ± 0.10	0.07 ± 0.02
**L304K**	5.2 ± 0.5	15.2 ± 3.1^a^	11.5 ± 1.8	0.34 ± 0.08	0.45 ± 0.08

Data obtained at 25 °C in 20 mM PIPES pH 7.0, 10 mM MgCl_2_.

^a^Values showing statistically significant differences from the WT, as determined by the one-way ANOVA test (*P* < 0.002; n = 3, confidence interval 95%).

### *Ca*FADS variants interacting with flavins and ATP

**Figure 3:**
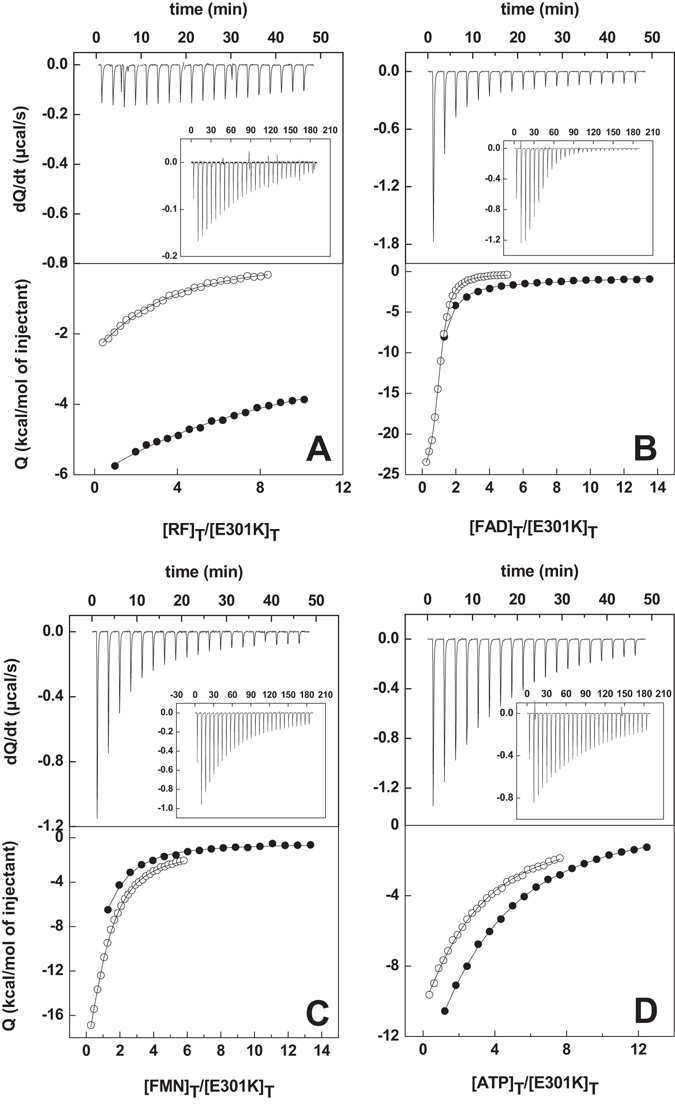
Calorimetric titrations of *Ca*FADS with: (**A**) RF, (**B**) FMN, (**C**) FAD and (**D**) ATP. Upper panels show thermograms for E301K and WT (inset) and lower panels show the corresponding binding isotherms with normalized integrated heats for E301K (●) and WT (○). Experiments carried out in 20 mM PIPES, 10 mM MgCl_2_, pH 7.0, at 25 °C.

Isothermal titration calorimetry (ITC) was used to determine the binding parameters that describe the formation of *Ca*FADS:flavin complexes (Table 3, Figure 3). Upon titration with RF, a single binding site was detected for K202A, E203A, F206W, V300A and L304A *Ca*FADSs. Thermograms for F206A and V300K variants reveal that their interaction with RF, although observed in difference spectra (Figure SP3), is weak or occurs with a very low enthalpy change. The remaining variants exhibit the same binding stoichiometry as the native protein (two RF sites) but with altered affinities. F206K and D298A *Ca*FADSs present slightly higher affinity for RF (*K*_d_ values only 2- and 4.5-fold lower than WT, respectively), while replacement of E301 by Lys decreases the affinity (Figure 3A, Table 3).

**Table 3:** Binding parameters for the interaction of WT and mutated *Ca*FADSs with RF, FMN, FAD and ATP, as determined by ITC (n = 3; means ± SD).

	*K*_d_ (µM)					
	FADS:RF	FADS:FMN	FADS:FAD	FADS:ATP	FADS:ADP:FMN	
				10 mM MgCl_2_	FMNAT site	RFK site
**WT** ^**a**^	24.1 (2) ± 3.6	7.8 (1) ± 0.9	0.74 (1) ± 0.10	30.2 (2) ± 4.5	0.04 ± 5 10^−3^	0.90 ± 0.10
**K202A**	31.4 (1) ± 4.7	12.2 (≪1) ± 1.0	6.4 (≪1) ± 1.0	64.5 (1) ± 16.9	0.01 ± 1.5 10^−3^	1.4 ± 0.3
**E203A**	10.3 (1) ± 2.3	7.2 (1) ± 1.6	61.8 (1) ± 15.2^c^	43.4 (2) ± 9.3	0.94 ± 0.20	9.12 ± 1.4^c^
**F206A**	n.d.^b^	6.8 (1) ± 3.8	1.9 (≪1) ± 0.1	15.3 (2) ± 2.4	1.2 ± 0.7^c^	9.71 ± 2.7^c^
**F206K**	7.6 (2) ± 2.5	3.1 (≪1) ± 0.5	11.5 (1) ± 1.7	29.1 (2) ± 8.0	0.10 ± 0.03	0.39 ± 0.06
**F206W**	10.3 (1) ± 4.0	3.6 (≪1) ± 1.1	0.68 (≪1) ± 0.30	26.5 (2) ± 11.0	0.05 ± 0.01	0.85 ± 0.30
**D298A**	5.3 (2) ± 1.1	18.8 (1) ± 2.9^c^	3.0 (≪1) ± 0.3	32.9 (2) ± 2.7	1.2 ± 0.2^c^	12.09 ± 2.1^c^
**D298E**	17.9 (2) ± 8.5	19.1 (1) ± 1.8^c^	3.7 (≪1) ± 0.2	46.2 (2) ± 14.5	1.4 ± 0.3^c^	3.4 ± 0.6
**V300A**	22.6 (≪1) ± 1.7	2.5 (≪1) ± 0.5	2.2 (≪1) ± 1.0	31.6 (2) ± 9.1	0.10 ± 0.005	3.1 ± 0.5
**V300K**	n.d.^b^	n.d.^b^	4.0 (1) ± 0.5	15.3 (2) ± 2.4	0.22 ± 0.03	2.2 ± 0.4
**E301A**	58.0 (2) ± 8.4	5.9 (1) ± 0.7	2.4 (1) ± 0.9	60.7 (2) ± 9.5	0.35 ± 0.07	4.7 ± 0.6
**E301K**	142 (2) ± 42^c^	13.6 (1) ± 1.2	2.9 (1) ± 0.4	60.2 (2) ± 10.1	0.19 ± 0.04	13.2 ± 1.9^c^
**L304A**	1.2 (1) ± 0.4	18.3 (1) ± 0.8^c^	28.2 (1) ± 3.3^c^	17.6 (1) ± 1.0	0.51 ± 0.20	1.1 ± 0.3
**L304K**	40.6 (2) ± 7.7	37.1 (1) ± 3.0^c^	26.2 (1) ± 1.0^c^	39.6 (2) ± 2.5	1.7 ± 0.1^c^	2.2 ± 0.5

The stoichiometry for the interaction is shown in brackets. For those showing a stoichiometry of N = 2, data correspond to an average *K*_d_ (*K*_d,av_) of two independent binding sites. Data obtained at 25 °C in 20 mM PIPES pH 7.0, 10 mM MgCl_2_.

^a^Data from^16, 21^.

^b^Despite these variants exhibiting different spectra upon mixing with the ligand, no heat exchange was detected in the corresponding ITC titration, indicating a very low binding enthalpy.

^c^Values showing statistically significant differences from the WT, as determined by the one-way ANOVA test (*P* < 0.002; n = 3, confidence interval 95%).

When studying the interaction of these variants with FAD and FMN, several demonstrate low occupancy for either one or both of these flavins (Table 3), suggesting non-productive assemblies that block flavin access to their binding sites^19^. Differences in FMN affinity were observed, with relatively weaker interactions for D298 and L304 variants, and a very low enthalpy change for V300K. Most of the introduced mutations reduced FAD affinity, particularly E203A, L304A and L304K (*K*_d_ increases up to 80-fold relative to WT). In general, these differences in binding affinities reveal that the mutations modulate the interactions of flavins with their binding cavity in the FMNAT module.

When analyzing the interaction of the *Ca*FADS variants with ATP, in the presence of 10 mM MgCl_2_, a single binding site was observed for K202A and L304A, while the rest of the variants retained the two independent ATP binding sites of WT *Ca*FADS^16, 20, 21^. Mutations only produced minor effects in the average ATP affinity, with changes introduced at charged residues causing slight decreases and changes at some hydrophobic residues causing slight increases (Table 3). Thus, these mutated residues barely modulate ATP binding at either or both of the FMNAT or RFK modules.

**Figure 4:**
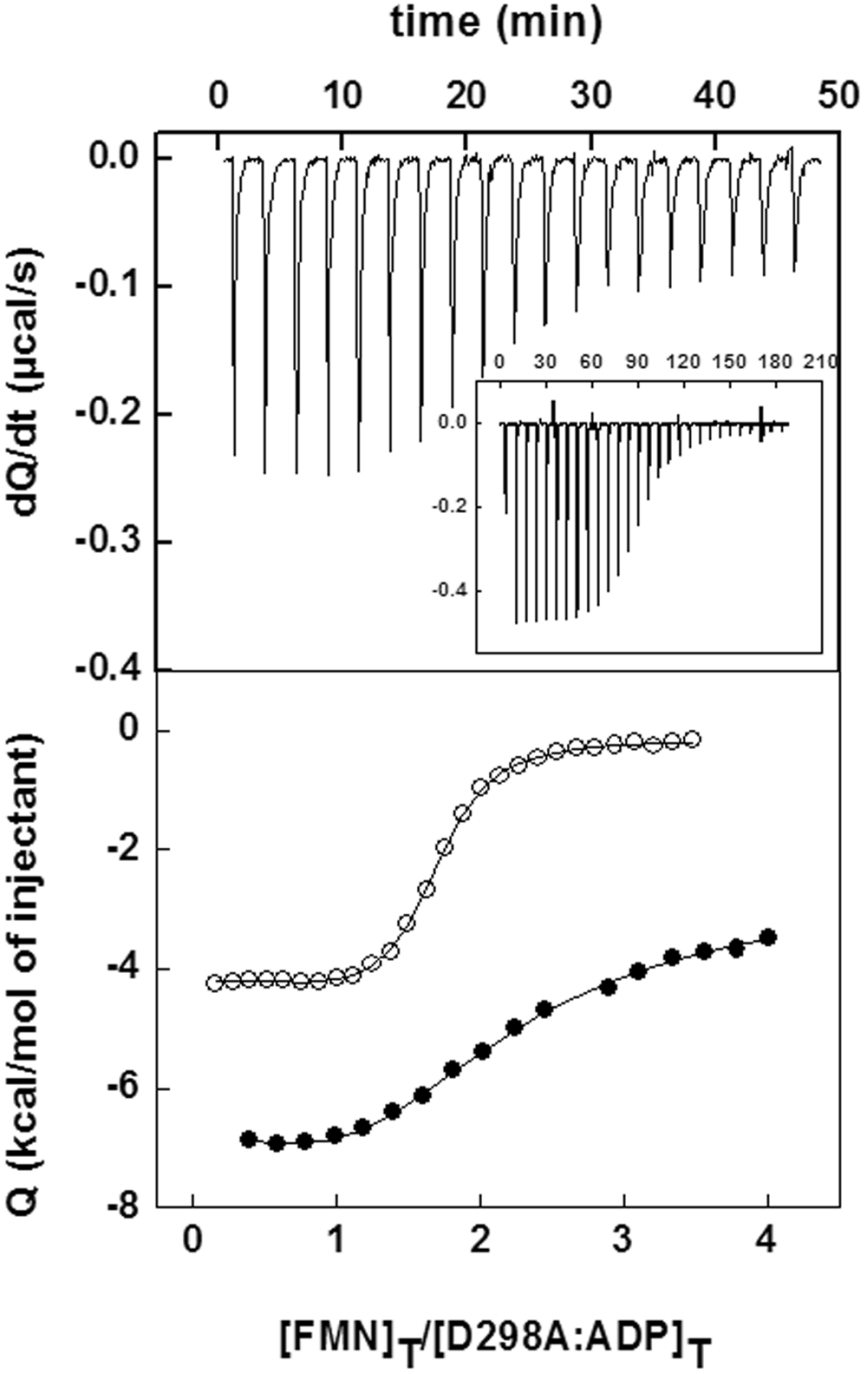
(**A**) Calorimetric titration of the preformed *Ca*FADS:ADP:Mg^2+^ complex with FMN. The upper panel shows thermograms for the D298A and WT (inset) titrations and the lower panel displays the corresponding binding isotherms with normalized integrated heats for D298A (●) and WT (○). Experiments carried out in 20 mM PIPES, 10 mM MgCl_2_, pH 7.0, at 25 °C.

We have also investigated the interaction of FMN with the preformed *Ca*FADS:ADP:Mg^2+^ complex. None of the mutations prevents the FMN binding to the two independent binding sites reported for WT^20^. In general, the mutations appear to disturb the FMN interaction at the high-affinity binding site, presumably the site in the FMNAT module, demonstrating significantly higher *K*_d_ values for F206A, D298A, D298E and L304K (Table 3). Only the E203A, F206A, D298A and E301K mutations significantly decrease the affinity of the second FMN-binding site, putatively in the RFK module (*K*_d_ 10–15-fold higher) (Figure 4, Table 3). These results suggest that E203, F206, D298, E301 and L304 modulate the interaction of the FMN substrate at both the RFK and FMNAT binding sites.

The large values of the enthalpic and entropic contributions reported for FMN, FAD and ATP binding to WT *Ca*FADS have been related to the formation of a large number of interactions, as well as the displacement of numerous well-ordered water molecules on the protein surface, as a consequence of both ligand binding and the assembly of the dimer of trimers^19^. The corresponding values for the mutants in this work are presented in Figures SP5 and SP6 and Tables SP1 and SP2. In short, these data indicate that these mutations noticeably modulate enthalpic and entropic contributions to ligand binding, with the mutations inducing, in general, the loss of favorable interactions at the FMNAT binding site and the formation of favorable interactions in the RFK module. These data further support the presence of differences between the conformations of the assemblies in these variants.

### Crystal Structure of the *Ca*FADS variants

The overall structures of F206W, D298E and E301A *Ca*FADSs are quite similar to that of the WT (A chains r.m.s.d. values 0.149, 0.152 and 0.251 Å, superimposing 274, 309 and 317 atoms, respectively). They all have two chains in the asymmetric unit and the relative positioning between the two modules of each protomer is identical to that of WT^6, 22^. All mutants contain residues 1 through 338, corresponding to the whole sequence, although in some high-mobility regions the lack of electron density prevents determining of the structural location of some residues. Thus, in the F206W structure, residues 200–204 in chain A and 201–202 in chain B from L1c, as well as residues 259–263 (in chain A) and 261–264 (in chain B) from L4c-FlapII, are not observed. The D298E structure lacks density for residues 259, 260, 261 and 262. The E301A structure shows all residues in chain A but lacks 260, 261 and 262 in chain B. All mutant structures contain, in addition, one sulfate ion and one pyrophosphate molecule, as ligands in each chain. The PISA server^23^ predicts a stable dimer of trimers assembly in solution for the three variants, with similar stability as the WT; in contrast, prediction of a single stable trimeric assembly is uncertain (Table SP3). Low B factors at residues forming the interface between modules of a single protomer do not predict hinge movements in any of the variants, similarly to that reported for the WT enzyme^6^.

**Figure 5:**
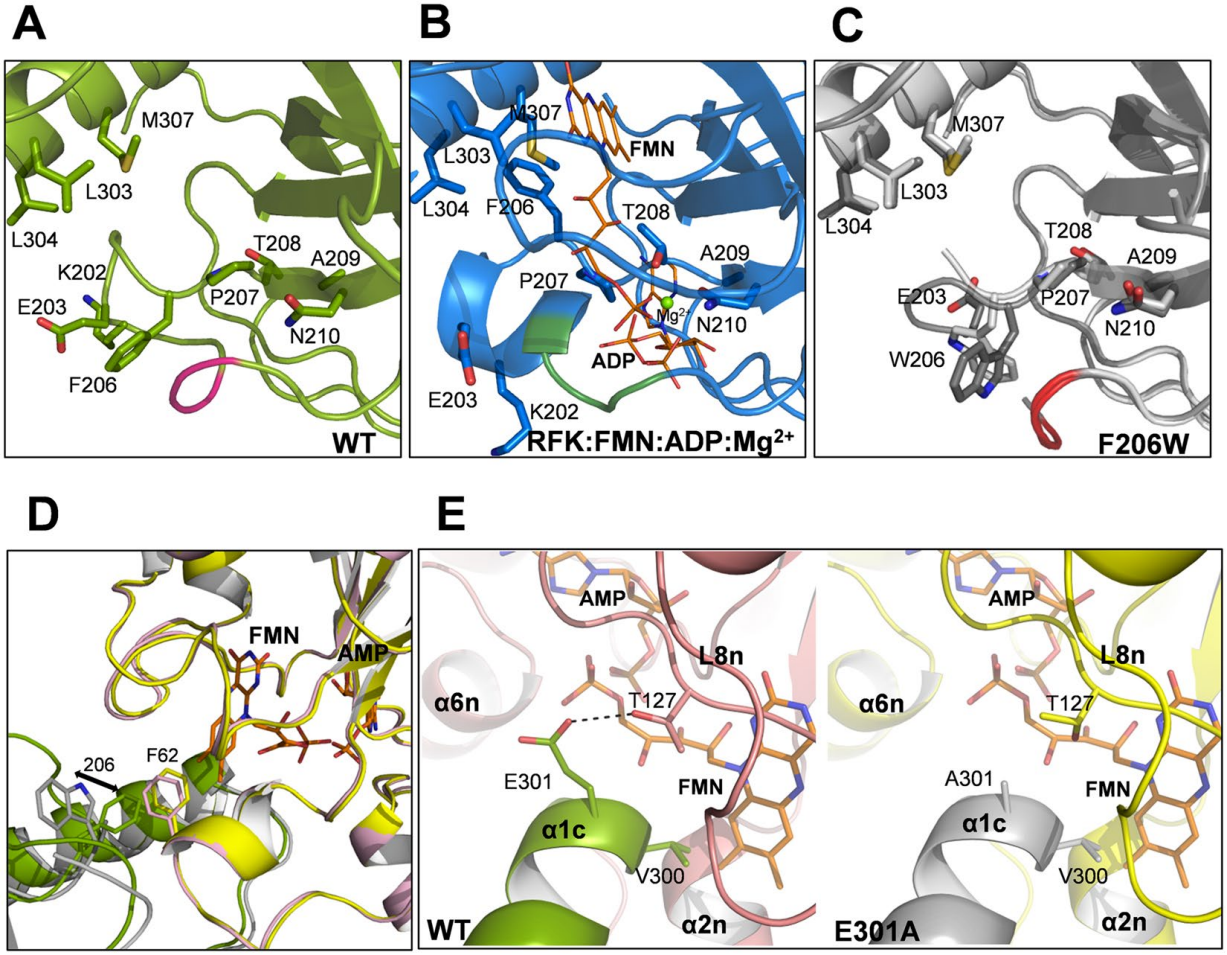
Structural analysis of *Ca*FADS variants. Detail of the RFK module around the F206 position in (**A**) WT *Ca*FADS (PDB code: 2x0k) (green), (**B**) the WT *Ca*FADS ternary complex with the reaction products (PDB code: 5a89) (blue) and (**C**) superposition of the two chains of the asymmetric unit (Chain A in light gray and Chain B in dark gray) in F206W *Ca*FADS with the two different conformations adopted by W206 (PDB code: 5fnz). Relevant residues are represented in sticks and CPK colored, and residues 197, 198 and 199 of L1c-FlapI are highlighted in pink, green and red, respectively. FMN and ADP ligands of the ternary complex are depicted as narrow sticks with orange carbons and the Mg^2+^ ion is shown as a green sphere. (**D**) Superposition of predicted macromolecular head-to-tail interfaces at the RFK and FMNAT modules of contiguous protomers within each trimer in WT *Ca*FADS (modules in green and pink, respectively) and in F206W *Ca*FADS (modules in gray and yellow, respectively). Residues 62 and 206 are shown in colored sticks. An arrow indicates the change in side chain conformations at position 206 in the mutant. (**E**) Detail of the predicted trimer head-to-tail interface region around position 301 in WT (left panel, RFK module in green and FMNAT module in pink) and E301A (right panel, RFK module in gray and FMNAT module yellow, respectively) *Ca*FADS variants. In (**D**,**E**) FMN and AMP ligands in the FMNAT module have been modelled as previously described and are shown in sticks colored with carbons in orange^6^.

For the F206W variant, the side chain of the introduced tryptophan was refined in slightly different conformations for each of the two molecules of the asymmetric unit. Both differ from those of F206 in the WT structure and in the substrate-bound complex (Figure 5A–C). Moreover, residues 197–199 on L1c-FlapI of F206W *Ca*FADS were displaced relative to those of the WT (Figure 5A,C), contributing to the opening of the cavity where ADP binds in the RFK module (Figure 5B). Noticeably, these residues immediately precede the region that stabilizes a 3_10_ α-helix (199–204) in the ternary complex structure of the *Ca*FADS RFK module that contains the reaction products, FMN:ADP:Mg^2+^ (Figure 5B). The PISA server predicts a dimer of trimers for the F206W structure, in which the orientations of F62 and W206 side chains from the two neighboring protomers differ from the WT, and the distance between the aromatic rings increases by 1.34 Å (Figure 5D).

No differences were observed in the D298E *Ca*FADS structure relative to that of WT. Replacement of D298 with Glu allows carboxylic oxygen atoms to maintain their bond with T165 of the neighboring protomer in the trimer (Figure SP7). Finally, no conformational changes were observed in L6c or in the α1c helix, which contains the E301A mutation, when comparing this mutant’s structure to that of the WT. However, the H-bond between E301 of one protomer and T127 on loop L8n of the neighboring protomer, which is predicted for the WT dimer of trimers, is not expected in this case (Figure 5E).

## Discussion

All studied variants with mutations in L1c-FlapI, L6c and helix α1c are active, as well as able to bind ligands and to stabilize transient quaternary assemblies. The lack of drastic changes prevents us from making conclusions regarding channeling between modules of different protomers, a question previously raised for the *Ca*FADS dimer of trimers^6^. Despite the fact that the effects are not drastic, point mutations, particularly in L1c-FlapI and helix α1c, affect the equilibria between *Ca*FADS monomeric/oligomeric assemblies, as well as kinetic and ligand binding parameters. All mutants also maintain the WT inhibition profile induced by an excess of the RF substrate. This is a critical aspect of the regulation of the RFK activity in *Ca*FADS, to which product inhibition might also contribute^16, 21^. Work is underway to understand the different levels of inhibition occurring in this enzyme. Nevertheless, those studies are beyond the scope of the present work, since the variants presented here do not demonstrate statistically significant differences in *K*_i_^RF^ relative to the WT (Table 1). Therefore, in this work, we emphasize interpreting the effects of mutations on the kinetic and binding parameters in view of the available structural information.

Mutations in L1c-FlapI (K202, E203 and F206) modulated the kinetic parameters for the RFK activity of *Ca*FADS, as well as ligand binding parameters in the C-terminal module (Tables 1, 3 and SP1). Therefore, although the relevant residues are not directly involved in catalysis, they somehow contribute to achieving the appropriate RFK catalytic geometry. This relationship is consistent with the recently reported structure for the *Ca*FADS RFK:FMN:ADP:Mg^2+^ ternary complex (Figure 1B)^9^. Complex formation induces conformational changes in L1c-FlapI, β2c and the 207-PTAN-210 motifs, which displace Cαs of K202 and E203 by 8.1 and 7.2 Å, respectively. Concomitantly, the F206 side chain (highly conserved in FADSS^24^) moves more than 7 Å to stack against M307 of helix α1c and the isoalloxazine ring of the flavin ligand^6, 9^ (Figure 1B). In these variants, *K*_m_^FMN^ for the FMNAT activity, as well as thermodynamic parameters for ligand binding at the FMNAT module (Tables 2, 3 and SP1), reveal the importance of L1c-FlapI residues in FMN and FAD binding in the N-terminal module. Moreover, the low fraction of protein that is capable of FAD and FMN binding that some of these variants exhibit is consistent with the coexistence of competent conformations in equilibrium with non-competent assembles that hinder flavin binding (Table 3)^19^. Furthermore, the strong effect of the E203A and F206A mutations on FMN binding at the FMNAT site in the *Ca*FADS:ADP:Mg^2+^ preformed complex (Tables 3 and SP2) align with the observation that their monomer/oligomer ratios after purification differ from that of the WT (Figure SP1A). In the WT *Ca*FADS dimer of trimers, K202 and E203 stabilize salt-bridges with E130 and N131 from loop L8n of the neighboring protomer within the trimer, while F206 interacts with F62 of helix α2n (Figure 1A)^6^. The mutation of K202 and E203 to Ala would prevent formation of the salt-bridges, while substitution of F206 by Ala or Lys will abrogate its hydrophobic interaction with F62. With the elimination of such interactions, the shape of the isoalloxazine hydrophobic cavity in the FMNAT site should change, as found when evaluating this cavity in the dimer of trimers assembly for the F206W variant (Figure 5D). This conservative substitution apparently reduces the amount of oligomeric assemblies able to bind FMN and FAD at the FMNAT site, relative to the WT, an effect that is clearly overcome in the ternary complex (Table 3). Altogether, these data indicate that L1c-FlapI and, particularly, F206, contribute to modulating the conformations and the catalytic properties of quaternary assemblies of *Ca*FADS, although the individual residues studied here are neither critical for catalysis nor key determinants in assembling the protein structure.

Mutations in L6c (D298) and the contiguous N-terminal helix α1c (V300, E301 and L304) considerably modulate *k*_cat_ and *K*_m_^RF^ for the RFK activity as well as *K*_m_^FMN^ for the FMNAT activity (Tables 1 and 2). In general, the mutations decreased the affinity for FAD and FMN, and V300A and D298 variants also show low flavin occupancy (Table 3). These results, together with the observed changes in the thermodynamic parameters (Figures SP5 and SP6, Tables SP1 and SP2), indicate that these residues contribute to the binding of flavins in the FMNAT module as well as to the stabilization of quaternary assemblies. These mutations also affect the flavin binding in the RFK module, as seen in the decrease of the FMN affinities of their *Ca*FADS:ADP:Mg^2+^ complexes (Table 3). These effects (Tables 1 and 3) are consistent with recent studies that show that ligand binding and catalysis at the RFK site triggers dramatic conformational changes in L1c-Flap1, L4c-FlapII, L6c and helix α1c (Figure 1B)^9^. Residues here mutated do not directly interact with the flavin substrate, but L6c and helix α1c regions are part of the hydrophobic core that closes the *re*-face of the isoalloxazine ring (Figure 5B). In addition, L6c and the N-terminal end of helix α1c also contribute to closing the FMNAT flavin-binding site of the neighboring protomer within the *Ca*FADS trimer (Figures 1A and 5E). In particular, D298 establishes H-bonds with T165 at α1n of the adjacent protomer^6^, a residue reported as critical for substrate binding and FMN adenylylation (Figure SP7)^21^. D298 replacement by Ala would prevent this interaction, but in the D298E mutant, the introduced Glu can still retain it (Figure SP7). However, the presence of more than one orientation of carboxylic oxygens suggests that alterations to the FMNAT ligand-binding cavity within the dimer of trimers (Figure SP7) is a factor that determines the low flavin occupancy in the FMNAT sites of these variants (Table 3).

Among the residues mutated in helix α1c, only V300 is predicted to directly interact with the FMN isoalloxazine ring (1.38 Ǻ) in the FMNAT module of the adjacent protomer^6^ (Figures 1A and 5E). Substitution of V300 by Ala or Lys will affect the hydrophobic interactions, which can explain the changes observed in FMN and FAD binding parameters (Tables 3 and SP1). E301 and L304 are predicted to interact with T127 and A132, respectively, of L8n of the neighboring protomer FMNAT site (Figures 1A and 5E)^6^. The lack of interactions between the side chain of residue 301 and T127 in the E301A mutant and the substitution of L304 by Ala would both affect the van der Waals contacts with L8n in the dimer of trimers, producing local effects at this loop that helps close the FMNAT substrate binding site. These mutations can also alter the positive end of the helix α1c dipole, which contributes to the stability of substrate phosphate groups and is located near the FMNAT substrate binding site (Figure 5E)^6^. Since ligand binding and catalysis in the RFK module also trigger the conformational change of L6c and the bending of the helix α1c^9^, it is very likely that interplay between RFK and FMNAT modules of protomers, within the trimer, will differ when introducing mutations at L6c and α1c (Figures 2 and SP4). However, we cannot further speculate until structures containing the FMNAT module in complex with ligands become available. All these structural considerations also explain the changes in kinetic and binding parameters of the FMNAT module for the *Ca*FADS mutants (Tables 1, 2 and 3), which suggest a different conformation for flavin binding, especially, for FMN (Table SP1 and Figure SP4).

Altogether, our data indicate that the secondary structural elements of the *Ca*FADS RFK module investigated herein tune protomer-protomer contacts within the trimer. This confirms the interplay between protein-protein assembly and catalysis, with a possible role of macromolecular interfaces contributing to flavin homeostasis^8^. In this direction, recent studies on the human FMNAT, FADS2, have proven direct delivery of the flavin cofactor to the recipient apoflavoprotein through heterologous protein-protein interactions^25^. If this mechanism also occurs in *Ca*FADS, we might envisage the possibility of macromolecular assemblies as part of a cell strategy to control the delivery of FMN and FAD to apoflavoproteins. However, the study of such new strategies lies beyond this work.

## Conclusion

The individual mutations analyzed here, in loops L1c-FlapI, loop L6c and helix α1c of the RFK module of *Ca*FADS, are not unique determinants in the formation of quaternary assemblies, but modulate oligomerization profiles, binding and kinetic parameters for RFK and FMNAT activities. These data indicate that the mutated residues modulate the conformations and geometries of the formed assemblies. The nature of the mutated side chains influences the conformations of structural elements that contain them. In turn, these secondary structural elements modulate the packing architecture within quaternary assemblies, as well as ligand binding and kinetic parameters. In this context, the formation of transient oligomeric structures during the catalytic cycle of *Ca*FADS might be used to control the interplay between flavin synthesis and delivery.

## Methods

### Biological material

pET28a-*Ca*FADS plasmids containing the K202A, E203A, F206A, F206K, F206W, D298A, D298E, V300A, V300K, E301A, E301K, L304A and L304K mutations were obtained from *Mutagenex Inc.* Proteins were overexpressed in BL21(DE3) *E. coli* cells and purified, following a modification of the protocol previously reported, which consists of 20% ammonium sulfate fractionation, followed by sequential phenyl-sepharose and DEAE-cellulose chromatographies^20^. Protein purity was assessed by SDS-PAGE. Purified samples were dialyzed in 20 mM PIPES, pH 7.0, and quantified using the theoretical extinction coefficients, ε_279_ = 33.9 mM^−1^cm^−1^ for F206W and ε_279_ = 27.8 mM^−1^ cm^−1^ for WT and the rest of variants.

### Size-distribution analysis

A Superdex 200 10/300 GL column (*GE Healthcare Life Sciences*), previously equilibrated with 20 mM PIPES, 0.8 mM MgCl_2_, pH 7.0, and calibrated with the Gel Filtration Calibration Kit LMW (*GE Healthcare Life Sciences*), was used to separate species by size^6, 8^. Chromatograms were fit to a series of Gaussian functions (Origin 7.0, *OriginLab*) to determine the number of components, their proportions, and their estimated masses (Figure SP8)^8, 19^. Considering the difficulty to unequivocally correlate different quaternary organizations for *Ca*FADS with similar hydrodynamic radii but different volumes and shapes (such as dimers, trimers, tetramers or hexamers) with their elution volumes, averaged values have been used for peaks mainly considered as monomeric species or oligomeric assemblies^8, 19^.

To evaluate the effects of ligands in the formation and dissociation of protein assemblies, monomeric and oligomeric fractions of the freshly purified variants were first separated using the Superdex 200 10/300 GL column. Then, samples containing either monomers or oligomers were incubated for 10 min at room temperature with either only buffer (20 mM PIPES, 0.8 mM MgCl_2_, pH 7.0), or with buffer and the products of RFK activity (FMN and ADP). After incubation samples were again passed again through the gel filtration column to remove ligands. Oligomers and monomers were recovered and their relative populations were calculated.

### Spectral analysis

Circular dichroism (CD) spectra were recorded with a Chirascan spectropolarimeter (*Applied Photophysics Ltd.*) at 25 °C as previously described^16, 20, 21^. Samples containing 5 µM *Ca*FADS in 5 mM PIPES, 10 mM MgCl_2_, pH 7.0 and 20 µM *Ca*FADS in 20 mM PIPES, 10 mM MgCl_2_, pH 7.0 were used in the far-UV (cuvette path length, 0.1 cm) or near-UV CD (0.4 cm), respectively. Difference spectroscopy measurements were carried out in 20 mM PIPES, 10 mM MgCl_2_, pH 7.0, with saturating concentrations of ligands^16, 20, 21^.

### Qualitative detection of RFK and FMNAT activities

RFK and FMNAT activities were qualitatively assayed by separating flavins from reaction mixtures by TLC on Silica Gel SIL-G-25 plates (20 × 20 cm, thickness 0.25 mm), as previously described^24^. The 150 μl volumes of reaction mixtures containing 50 μM RF or FMN, 0.2 mM ATP, 10 mM MgCl_2_, and ~200 nM of *Ca*FADS (a mixture of oligomeric species) in 20 mM PIPES, pH 7.0, were incubated for 5, 30 or 120 min at 37 °C. Reactions were stopped by boiling the preparations for 5 min. Flavin TLC spots were examined by monitoring their fluorescence under UV light.

### Steady-state kinetics parameters for the RFK and FMNAT activities

The *Ca*FADS RFK activity was measured at 25 °C in 500 µl of 20 mM PIPES, 0.8 mM MgCl_2_, pH 7.0, containing 0.5–45 µM RF and 10–500 µM ATP. Reactions were initiated by addition of ~20 nM of the monomeric enzyme. After 1 min incubation at 25 °C, the reactions were stopped by boiling the mixtures for 5 min. The flavin composition in the supernatant was determined using an Alliance HPLC system (*Waters*), equipped with a 2707 autosampler and an HSST3 column (4.6 × 150 mm, 3.5 µm, *Waters*), preceded by a precolumn (4.6 × 20 mm, 3.5 µm, *Waters*). Flavins (FMN or FAD) produced from RF were quantified using their corresponding standard curves, as previously described^21^.

The *Ca*FADS FMNAT activity was fluorometrically measured using a continuous assay. Measurements were performed in a final volume of 1 ml in 20 mM PIPES, 10 mM MgCl_2_, pH 7.0, containing 0–15 µM FMN, 0–400 µM ATP and ~40 nM of monomeric enzyme (higher concentrations were used for some variants) at 25 °C. A Cary Eclipse spectrophotofluorometer with excitation and emission wavelengths of 420 nm and 530 nm, respectively, was used. FAD and FMN fluorescence were individually calibrated using standard solutions and the data were analyzed as previously described^16^.

The kinetic data obtained for one substrate at saturating concentrations of the second substrate (as nmol of flavin transformed *per* min) were interpreted using the Michaelis-Menten kinetic model, obtaining *k*_cat_ and *K*_m_ with errors of ±10%. When an excess of RF inhibited the RFK activity, the experimental data were interpreted through a model describing the substrate inhibition of a bi-substrate mechanism^26^. In these situations, the errors in apparent *K*_m_ and *k*_cat_ (^app^ *K*_m_ and ^app^ *k*_cat_) increased as the inhibition constant (*K*_i_) approached *K*_m_^S^. Experiments were performed in triplicate.

### Isothermal titration calorimetry (ITC)

Measurements were performed using an AutoITC200 calorimeter (*MicroCal*), thermostated at 25 °C. Typically, 200 µM RF, FMN or FAD and 300 µM ATP solutions were used to titrate ~20 µM of monomeric *Ca*FADS in a 200 µl cell volume. Ligand and *Ca*FADS were dissolved in 20 mM PIPES, 10 mM MgCl_2_, pH 7.0, and degassed prior to titration. Up to 19 injections of 2 µl each were added to the sample cell and mixed via the rotating (1000 rpm) stirrer. Similar protein concentrations in the calorimetric cell were employed in all experiments to guarantee the same oligomerization state of each variant at all times^16^.

The association constant (*K*_a_), the enthalpy change (Δ*H*) and the stoichiometry (N), or their average values, were obtained through non-linear regression of the experimental data to a home-derived model for one or two independent binding sites; the regression was implemented in Origin 7.0 (*OriginLab*)^21^. The dissociation constant (*K*_d_), the free energy change (Δ*G*), and the entropy change (Δ*S*) were obtained from basic thermodynamic relationships. Experiments were performed in duplicate or triplicate. Errors in the measured parameters (±15% in *K*_d_ and ±0.3 kcal/mol in Δ*H* and −TΔ*S*) were larger than the standard deviation between replicates and the numerical error after the fitting analysis.

### Crystal growth, data collection and structure refinement of F206W, D298E and E301A *Ca*FADSs

Samples of all *Ca*FADS variants were dialyzed in 40 mM phosphate buffer, pH 6.8, and concentrated to 10 mg/ml. Crystallization conditions were similar to those previously used for the native protein, mixing a volume of 1.5 M Li_2_SO_4_ and 0.1 M HEPES/NaOH, pH 7.5, with the same volume of protein solution^22^. Crystals were cryoprotected with solutions containing 50% reservoir solution and 50% saturated Li_2_SO_4_ solution. Diffraction data sets were collected on the ID14-1 beamline at the European Synchrotron Radiation Facility (ESRF, Grenoble). Data sets were collected at 100 K using a wavelength of 0.93340 Å and processed, scaled and reduced with XDS^27^ and SCALA^28^ from the CCP4 package (Collaborative Computational Project, Number 4, 1994). MOLREP^29^ from CCP4 was used to solve all the structures with the native *Ca*FADS structure (PDB ID 2x0k) as a search model. Refinements were performed automatically by Refmac-5^30^ from CCP4 and manually by COOT^31^. SFCHECK^32^, PROCHECK^33^ and MOLPROBITY^34^ were used to assess and validate final structures.

Collection and structural data summarized in Table SP4 indicate that the F206W, D298E and E301A *Ca*FADS crystals belong to the same space group as those reported for WT FADS, also possessing similar unit cell dimensions and asymmetric unit compositions^22^. The PISA server^23^ was used to assess the oligomeric states of variants which might be consistent with their crystal structures, as well as to identify putative macromolecular interfaces and the residues involved in the interactions. Coordinates have been deposited in the PDB with PDB IDs: 5fnz for F206W, 5fo0 for D298E and 5fo1 for E301A.

### Statistics

Results are expressed as the mean ± the standard deviation (SD) or as the mean ± the standard error (SE) of the regression. When indicated, one-way analysis of variance (ANOVA) was performed to determine statistical significance.

## Electronic supplementary material

Supplementary Material

## References

[1] Gabizon R, Friedler A (2014). Allosteric modulation of protein oligomerization: an emerging approach to drug design. Front Chem.

[2] Marsh JA, Teichmann SA (2014). Protein flexibility facilitates quaternary structure assembly and evolution. PLoS Biol.

[3] Marsh JA, Teichmann SA (2015). Structure, dynamics, assembly, and evolution of protein complexes. Annu Rev Biochem.

[4] Korennykh AV (2011). Cofactor-mediated conformational control in the bifunctional kinase/RNase Ire1. BMC Biol.

[5] Matthews, J. M. *Protein dimerization and oligomerization in biology* (Springer Science & Business Media, 2012).

[6] Herguedas, B., Martinez-Julvez, M., Frago, S., Medina, M. & Hermoso, J. A. Oligomeric state in the crystal structure of modular FAD synthetase provides insights into its sequential catalysis in prokaryotes. *J Mol Biol* **400**, 218–230, doi:S0022-2836(10)00497-3 (2010). 10.1016/j.jmb.2010.05.018 20471397

[7] Wang W, Kim R, Yokota H, Kim SH (2005). Crystal structure of flavin binding to FAD synthetase of Thermotoga maritima. Proteins.

[8] Marcuello, C., Arilla-Luna, S., Medina, M. & Lostao, A. Detection of a quaternary organization into dimer of trimers of *Corynebacterium ammoniagenes* FAD synthetase at the single-molecule level and at the in cell level. *Biochim Biophys Acta* **1834**, 665-676, doi:S1570-9639(12)00293-2 (2013). 10.1016/j.bbapap.2012.12.013 23291469

[9] Herguedas B (2015). Structural insights into the synthesis of FMN in prokaryotic organisms. Acta Crystallogr D Biol Crystallogr.

[10] Yruela I, Arilla-Luna S, Medina M, Contreras-Moreira B (2010). Evolutionary divergence of chloroplasts FAD synthetase proteins. BMC Evol Biol.

[11] Nakamura Y, Nishio Y, Ikeo K, Gojobori T (2003). The genome stability in *Corynebacterium* species due to lack of the recombinational repair system. Gene.

[12] Seidel M, Alderwick LJ, Sahm H, Besra GS, Eggeling L (2007). Topology and mutational analysis of the single Emb arabinofuranosyltransferase of Corynebacterium glutamicum as a model of Emb proteins of Mycobacterium tuberculosis. Glycobiology.

[13] Grill, S., Busenbender, S., Pfeiffer, M., Kohler, U. & Mack, M. The bifunctional flavokinase/flavin adenine dinucleotide synthetase from Streptomyces davawensis produces inactive flavin cofactors and is not involved in resistance to the antibiotic roseoflavin. *J Bacteriol* **190**, 1546–1553, doi:JB.01586-07 (2008). 10.1128/JB.01586-07 PMC2258686 18156273

[14] Kearney EB, Goldenberg J, Lipsick J, Perl M (1979). Flavokinase and FAD synthetase from Bacillus subtilis specific for reduced flavins. J Biol Chem.

[15] Matern A, Pedrolli D, Großhennig S, Johansson J, Mack M (2016). Uptake and Metabolism of Antibiotics Roseoflavin and 8-Demethyl-8-Aminoriboflavin in Riboflavin-Auxotrophic Listeria monocytogenes. J Bacteriol.

[16] Serrano A (2013). Key residues at the riboflavin kinase catalytic site of the bifunctional riboflavin kinase/FMN adenylyltransferase from Corynebacterium *ammoniagenes*. Cell Biochem Biophys.

[17] Liuzzi, V. C. *et al*. Silencing of FAD synthase gene in Caenorhabditis elegans upsets protein homeostasis and impacts on complex behavioral patterns. *Biochim Biophys Acta* **1820**, 521–531, doi:S0304-4165(12)00030-X (2012). 10.1016/j.bbagen.2012.01.012 22306247

[18] Torchetti EM (2011). Human FAD synthase (isoform 2): a component of the machinery that delivers FAD to apo-flavoproteins. Febs J.

[19] Serrano A (2015). Quaternary organization in a bifunctional prokaryotic FAD synthetase: Involvement of an arginine at its adenylyltransferase module on the riboflavin kinase activity. Biochim Biophys Acta.

[20] Frago, S., Velázquez-Campoy, A. & Medina, M. The puzzle of ligand binding to Corynebacterium *ammoniagenes* FAD synthetase. *J Biol Chem* **284**, 6610–6619, doi:M808142200 (2009). 10.1074/jbc.M808142200 PMC2652324 19136717

[21] Serrano A, Frago S, Velázquez-Campoy A, Medina M (2012). Role of key residues at the flavin mononucleotide (FMN):adenylyltransferase catalytic site of the bifunctional riboflavin kinase/flavin adenine dinucleotide (FAD) Synthetase from Corynebacterium *ammoniagenes*. Int J Mol Sci.

[22] Herguedas, B., Martínez-Júlvez, M., Frago, S., Medina, M. & Hermoso, J. A. Crystallization and preliminary X-ray diffraction studies of FAD synthetase from Corynebacterium *ammoniagenes*. *Acta Crystallogr Sect F Struct Biol Cryst Commun* **65**, 1285–1288, doi:S1744309109044789 (2009). 10.1107/S1744309109044789 PMC2802882 20054130

[23] Krissinel, E. & Henrick, K. Inference of macromolecular assemblies from crystalline state. *J Mol Biol* **372**, 774–797, doi:S0022-2836(07)00642-0 (2007). 10.1016/j.jmb.2007.05.022 17681537

[24] Frago, S., Martínez-Júlvez, M., Serrano, A. & Medina, M. Structural analysis of FAD synthetase from Corynebacterium *ammoniagenes*. *BMC Microbiol* **8**, 160, doi:1471-2180-8-160 (2008). 10.1186/1471-2180-8-160 PMC2573891 18811972

[25] Giancaspero TA (2015). Remaining challenges in cellular flavin cofactor homeostasis and flavoprotein biogenesis. Front Chem.

[26] Leskovac, V. *Comprehensive enzyme kinetics* (Kluwer Adacemic/Plenum Publishers, 2003).

[27] Kabsch W (2010). Xds. Acta Crystallogr D Biol Crystallogr.

[28] Kabsch W (1988). Evaluation of single-crystal X-ray diffraction data from a position-sensitive detector. Journal of Applied Crystallography.

[29] Vagin A, Teplyakov A (1998). A translation-function approach for heavy-atom location in macromolecular crystallography. Acta Crystallogr D Biol Crystallogr.

[30] Murshudov GN, Vagin AA, Dodson EJ (1997). Refinement of macromolecular structures by the maximum-likelihood method. Acta Crystallogr D Biol Crystallogr.

[31] Emsley, P. & Cowtan, K. Coot: model-building tools for molecular graphics. *Acta Crystallogr D Biol Crystallogr* **60**, 2126–2132, doi:S0907444904019158 (2004). 10.1107/S0907444904019158 15572765

[32] Vaguine AA, Richelle J, Wodak SJ (1999). SFCHECK: a unified set of procedures for evaluating the quality of macromolecular structure-factor data and their agreement with the atomic model. Acta Crystallogr D Biol Crystallogr.

[33] Laskowski RA, MacArthur MW, Moss DS, Thornton JM (1993). PROCHECK: a program to check the stereochemical quality of protein structures. J. Appl. Cryst..

[34] Davis, I. W., Murray, L. W., Richardson, J. S. & Richardson, D. C. MOLPROBITY: structure validation and all-atom contact analysis for nucleic acids and their complexes. *Nucleic Acids Res* **32**, W615–W619, doi:10.1093/nar/gkh398 (2004). 10.1093/nar/gkh398 PMC441536 15215462

