# Supplementary material for: The trimer interface in the quaternary structure of the bifunctional prokaryotic FAD synthetase from Corynebacterium ammoniagenes
Source: Sci Rep. 2017 Mar 24;7:404. doi: 10.1038/s41598-017-00402-6 (PMC5428420; doi:10.1038/s41598-017-00402-6)
Supplement: Supplementary file 1 — Supplementary Material [file 41598_2017_402_MOESM1_ESM.pdf]

Supplementary Material

The trimer interface in the quaternary structure of the bifunctional prokaryotic FAD synthetase from *Corynebacterium ammoniagenes*

Ana Serrano[a,1,*], María Sebastián[a,*], Sonia Arilla-Luna[a], Silvia Baquedano[a], Beatriz Herguedas[a,2], Adrián Velázquez-Campoy[a,b,c], Marta Martínez-Júlvez[a] and Milagros Medina[a,‡]

[a]Departamento de Bioquímica y Biología Molecular y Celular, Facultad de Ciencias, and Institute of Biocomputation and Physics of Complex Systems, Universidad de Zaragoza, Spain

[b]Fundación ARAID, Diputación General de Aragón, Spain

[c]Aragon Institute for Health Research (IIS Aragon), Zaragoza, Spain

[‡]Correspondence to: Milagros Medina, Departamento de Bioquímica y Biología Molecular y Celular. Facultad de Ciencias, Universidad de Zaragoza. Pedro Cerbuna, 12. 50009 Zaragoza, Spain. Tel: +34976762476; Fax: +34976762123. E-mail: mmedina@unizar.es

[1]Present address: Centro de Investigaciones Biológicas, CSIC, Ramiro de Maeztu 9, E-28040 Madrid, Spain

[2]Present address: MRC Laboratory of Molecular Biology, Francis Crick Avenue, CB2 0QH, Cambridge, UK

*These authors contributed equally to this work.

Supporting figures

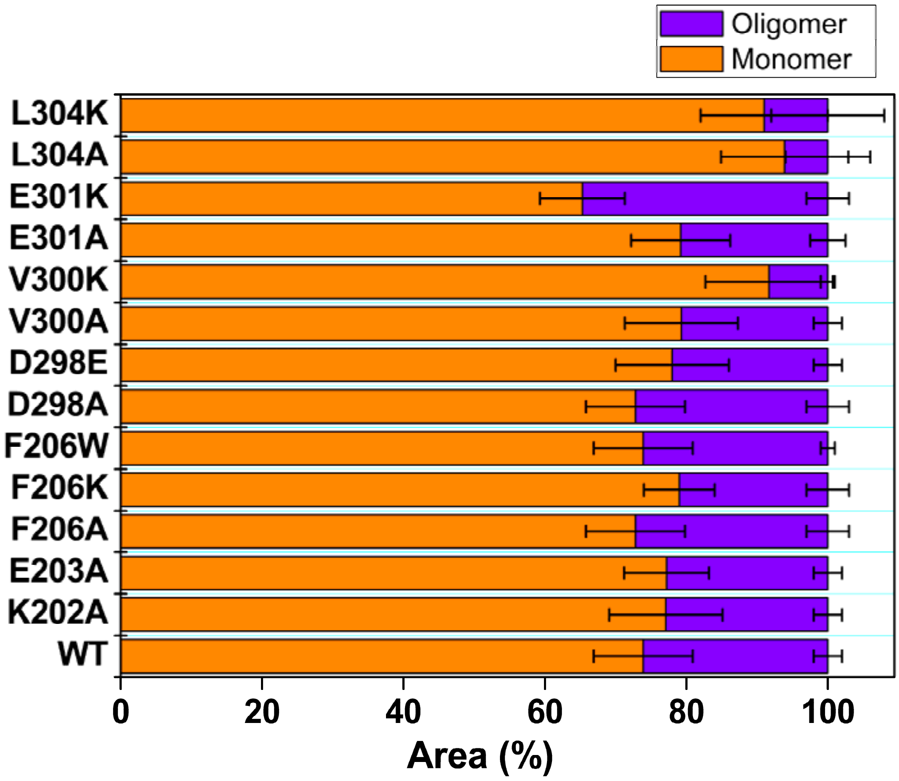

Figure SP1. Relative area of the monomeric and oligomeric species detected after passing the just purified variants of *Ca*FADS at ~20 µM through a Superdex^{TM} 200 10/300 GL column in 20 mM PIPES, 0.8 mM $MgCl_2$, pH 7.0. The one-way ANOVA test (n=3, confidence interval 95%) did not show statistically significant differences regarding the WT.

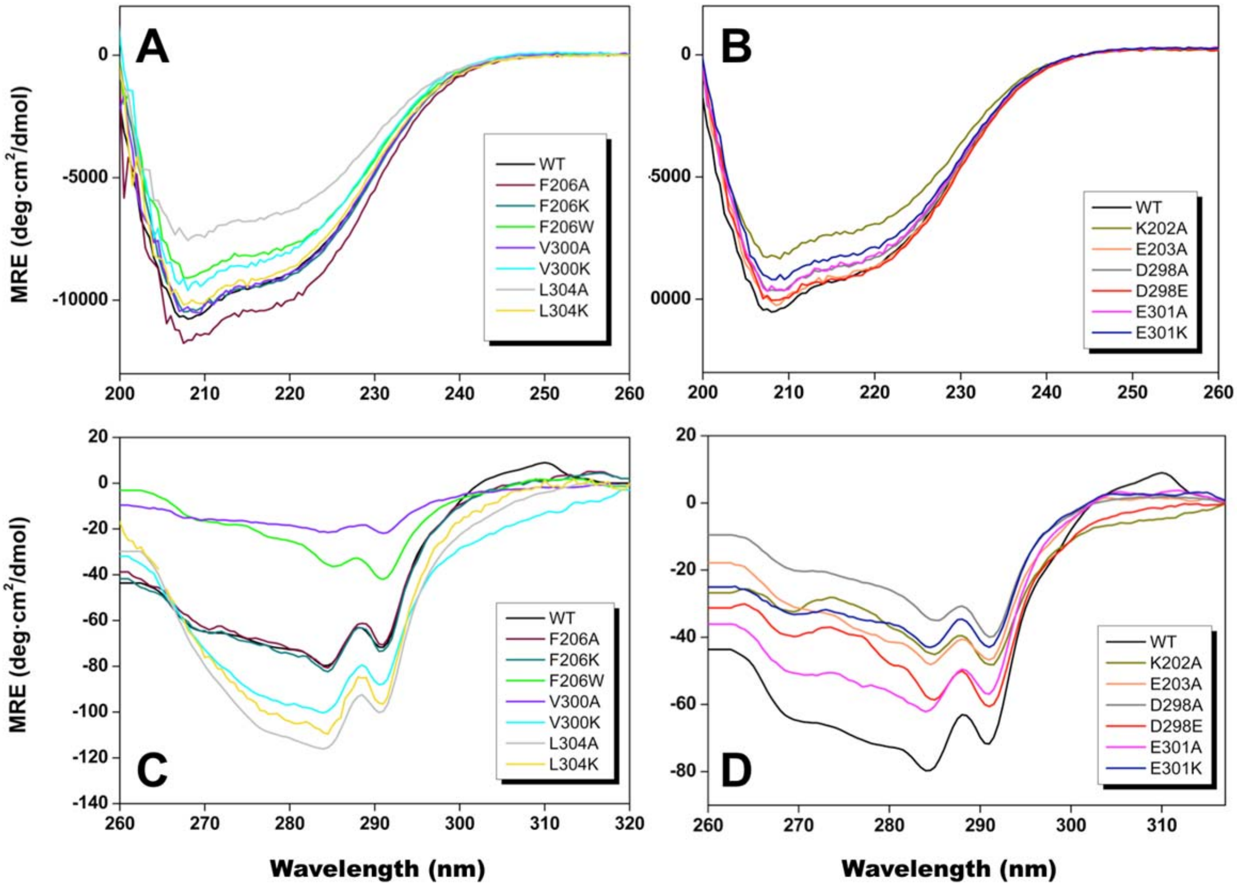

Figure SP2. Circular dichroism spectra (molar ellipticity *per* residue) (A and B) in the far-UV region and (C and D) in the near-UV region for the different *Ca*FADS variants. Spectra were recorded in 20 mM PIPES, 10 mM $MgCl_2$, pH 7.0 at 25 °C.

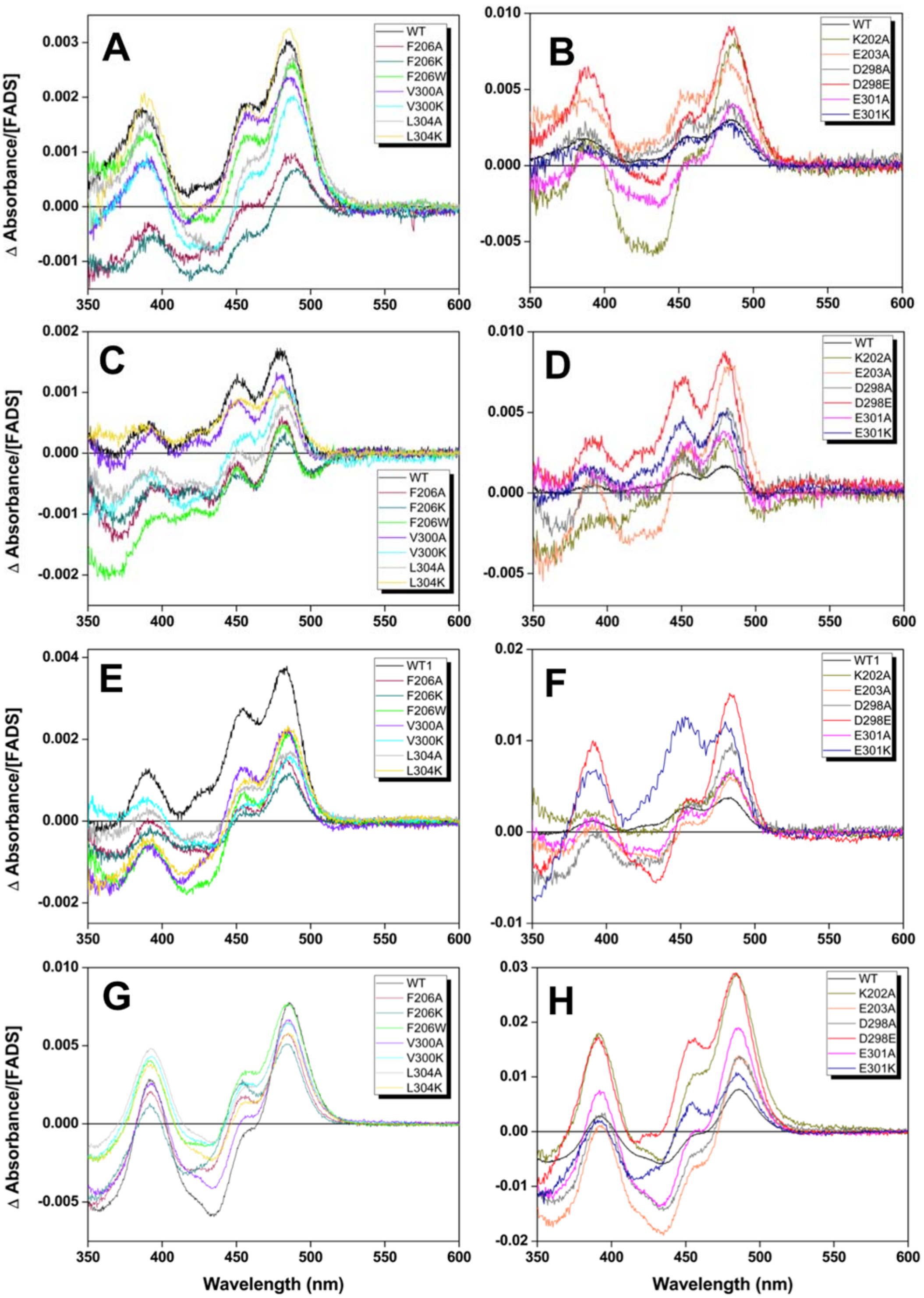

Figure SP3. Visible difference spectra obtained upon titration the different *Ca*FADS variants with saturating concentrations of (A and B) RF, (C and D) FAD, (E and F) FMN and (G and H) FMN in presence of ADP:Mg^{2+}. Spectra were recorded in 20 mM PIPES, pH 7.0 at 25 °C.

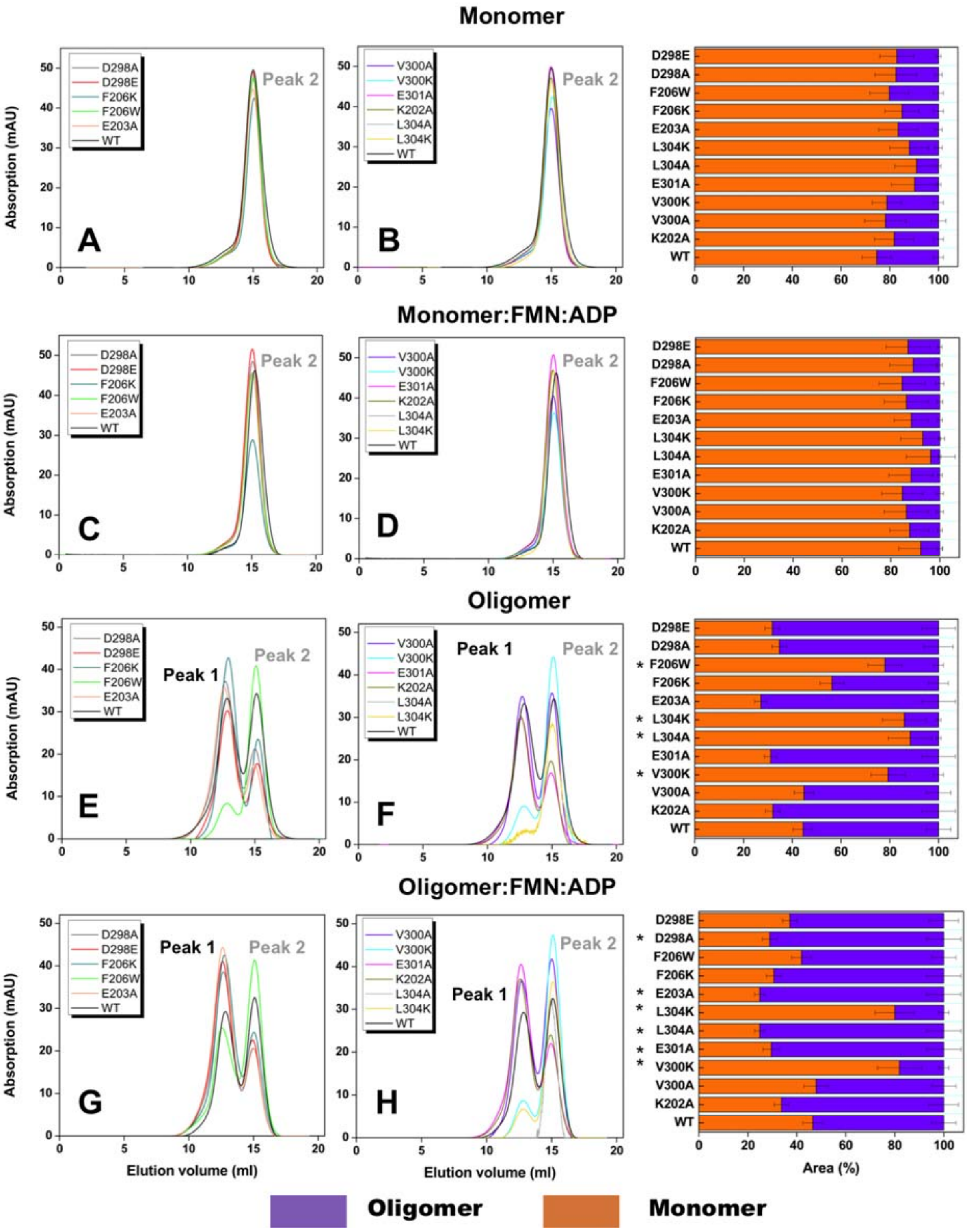

Figure SP4. Gel filtration elution profiles of purified monomeric and oligomeric fractions of the different *Ca*FADS variants under different conditions. (A and B) The peak corresponding to the monomeric form. (C and D) The band corresponding to the oligomeric forms. (E and F) The peak corresponding to the monomeric form incubated with 25 µM FMN and 200 µM ADP. (G and H) The band corresponding to the oligomeric forms incubated with 25 µM FMN and 200 µM ADP. Panels on the right show the corresponding percentages of monomer (peak 2) in orange bars and the bulk of oligomeric species (peak 1) in violet obtained from each chromatogram, with * indicating values showing statistically significant differences to the WT ones as determined by the one-way ANOVA test ($P < 0.002$; n=3, confidence interval 95%). All samples contained 15-20 µM of protein, 20 mM PIPES, 0.8 mM $MgCl_2$, pH 7.0, and where incubated 10 min at room temperature before passing through a Superdex™ 200 10/300 GL column in the same buffer.

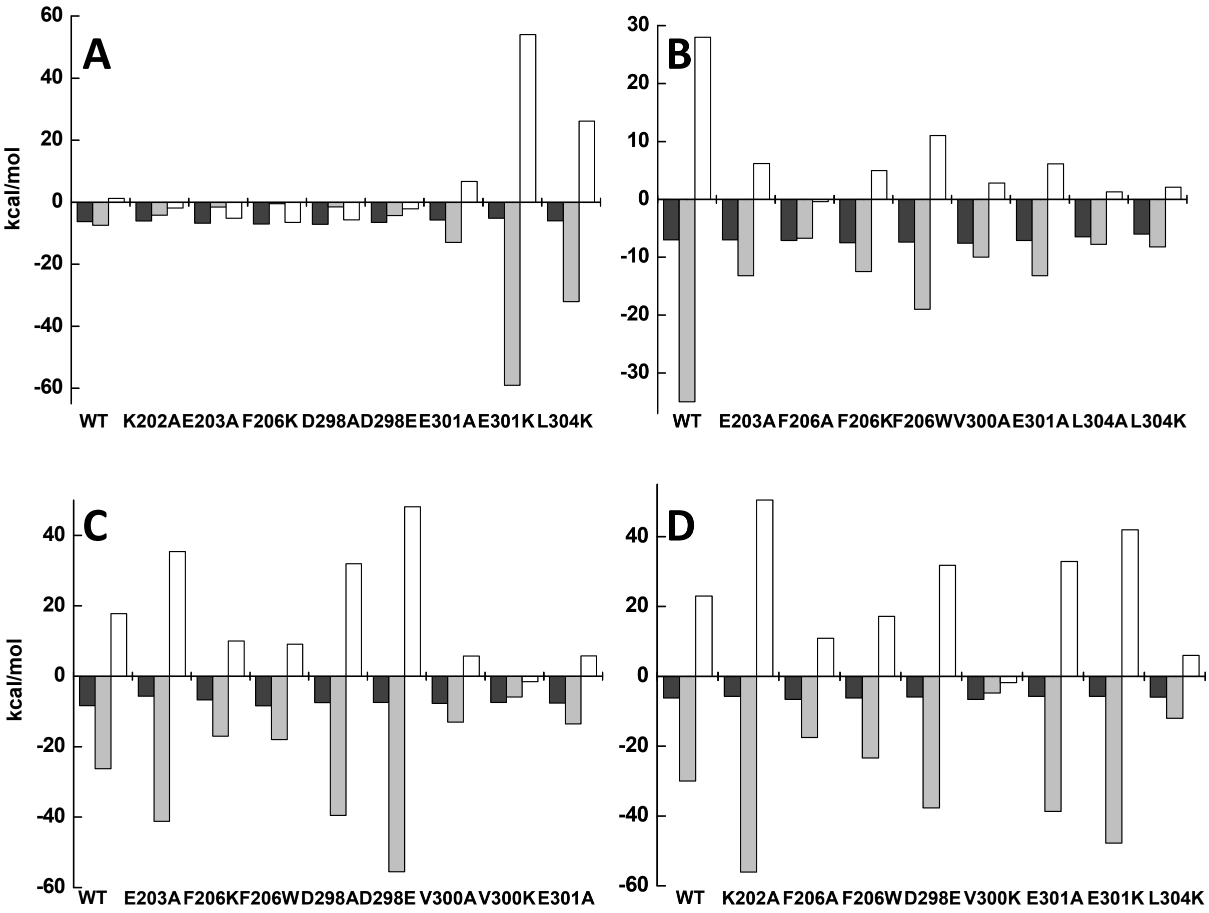

Figure SP5. Thermodynamic dissection of the interaction of the different *Ca*FADS variants with (A) RF, (B) FMN, (C) FAD and (D) ATP in 20 mM PIPES, 10 mM $MgCl_2$, pH 7.0, at 25 °C. The binding Gibbs energy (Δ*G*), enthalpy (Δ*H*), and entropy (-TΔ*S*) contributions to the binding are represented in dark grey, light grey and white bars, respectively.

These data show that the enthalpic contribution turns into more favorable and the entropic contribution into more unfavorable for variants at E301, particularly E301K, and for L304K when are compared with the WT (Panel A, Table SP1). For the rest of the variants RF binding is driven by the enthalpic contribution (more favorable than for WT *Ca*FADS) and by a favorable entropic contribution. FMN and FAD binding to WT *Ca*FADS is driven by a large enthalpic change with a big entropic cost (Panels B and C, Table SP1). All the mutations made the enthalpic contribution for the *Ca*FADS:FMN interaction considerably less favorable, while the entropic contribution was less unfavorable (even favorable for F206A) (Panel B, Table SP1). Larger differences in both magnitudes were observed for the FAD interaction regarding WT; more favorable enthalpic and less favorable entropic binding contributions for E203A, D298A and D298E, less favorable enthalpic and less unfavorable entropic contributions for F206K, F206W, V300A and E301A, and a favorable entropic contribution for V300K (Panel C, Table SP1), resulting in an obvious example of enthalpy-entropy compensation. Regarding ATP binding two patterns for thermodynamic parameters were observed regarding the WT. F206, V300 and L304 variants showed less favorable enthalpic and less unfavorable entropic contributions, while for the rest the magnitude of these parameters slightly increased (particularly for K202 and E301) (Panel D, Table SP1).

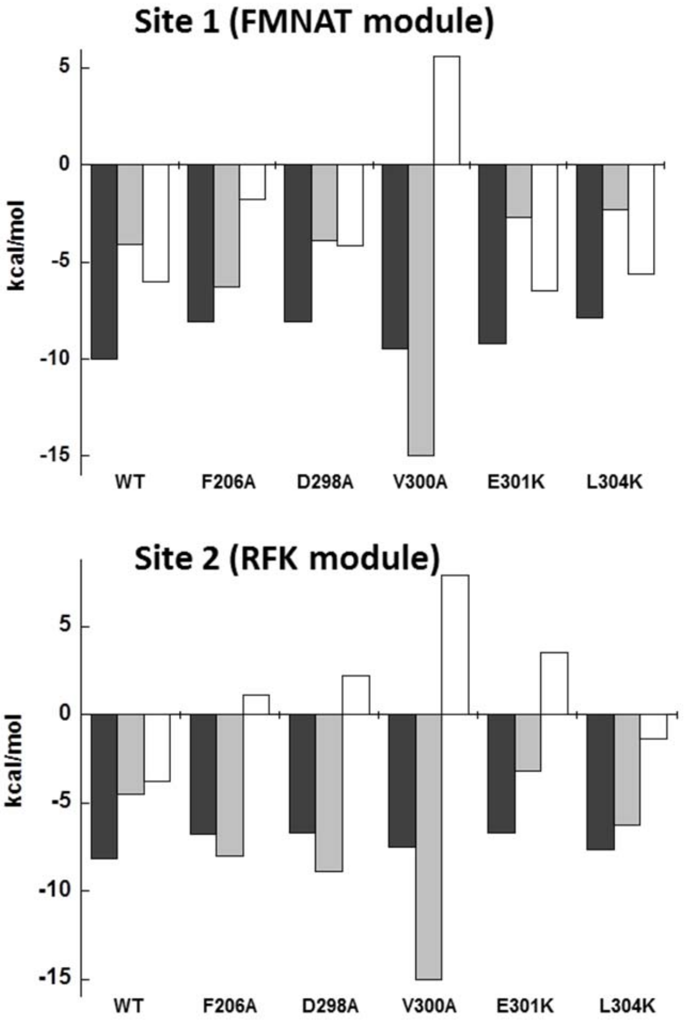

Figure SP6. Thermodynamic dissection of the interaction of the mutated *Ca*FADS:ADP preformed complex with FMN. Profiles at the two FMN binding sites are displayed separately. The binding Gibbs energy (Δ*G*), enthalpy (Δ*H*), and entropy (-TΔ*S*) contributions to the binding are represented in dark grey, light grey and white bars, respectively. Experiments carried out in 20 mM PIPES, 10 mM $MgCl_2$, pH 7.0, at 25 °C.

As reported for the WT [1,2], these data show that FMN binding to the preformed *Ca*FADS:ADP:Mg^{2+} complex is driven, in general, by favorable enthalpic and entropic contributions at both sites (Table SP2). This fact contrasts with the thermodynamic contributions driving FMN binding at the FMNAT module in the absence of ADP (compare Figure SP5B with Figure SP6 and Table SP1 with Table SP2). Changes in the magnitude of enthalpic and entropic contributions to the binding regarding the WT were particularly observed for V300A, for which the enthalpic contribution becomes more favorable and the entropic one becomes unfavorable at both sites (Table SP2). A similar change in the entropic contribution was observed for mutations F206A, D298A and E301K when analyzing FMN binding at the RFK module (Table SP2).

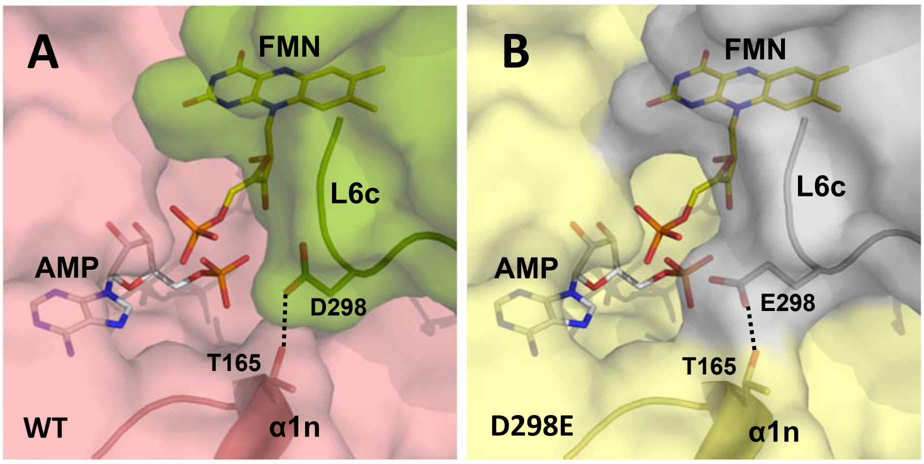

Figure SP7. Detail at the macromolecular interfaces predicted by PDBePISA among the RFK- and FMNAT-modules of contiguous protomers within the trimers for (A) WT (modules in green and pink, respectively) and D298E (in grey and yellow, respectively) *Ca*FADSs. Modules are represented as surfaces with cartoon representation of secondary structure elements implicated in the interaction of the side-chain at position 298 with T165 (shown as colored sticks). Theoretical position for FMN and AMP ligands in the FMNAT module is shown in yellow and grey CPK sticks, respectively, accordingly to the model previously proposed for the interaction to WT *Ca*FADS [3].

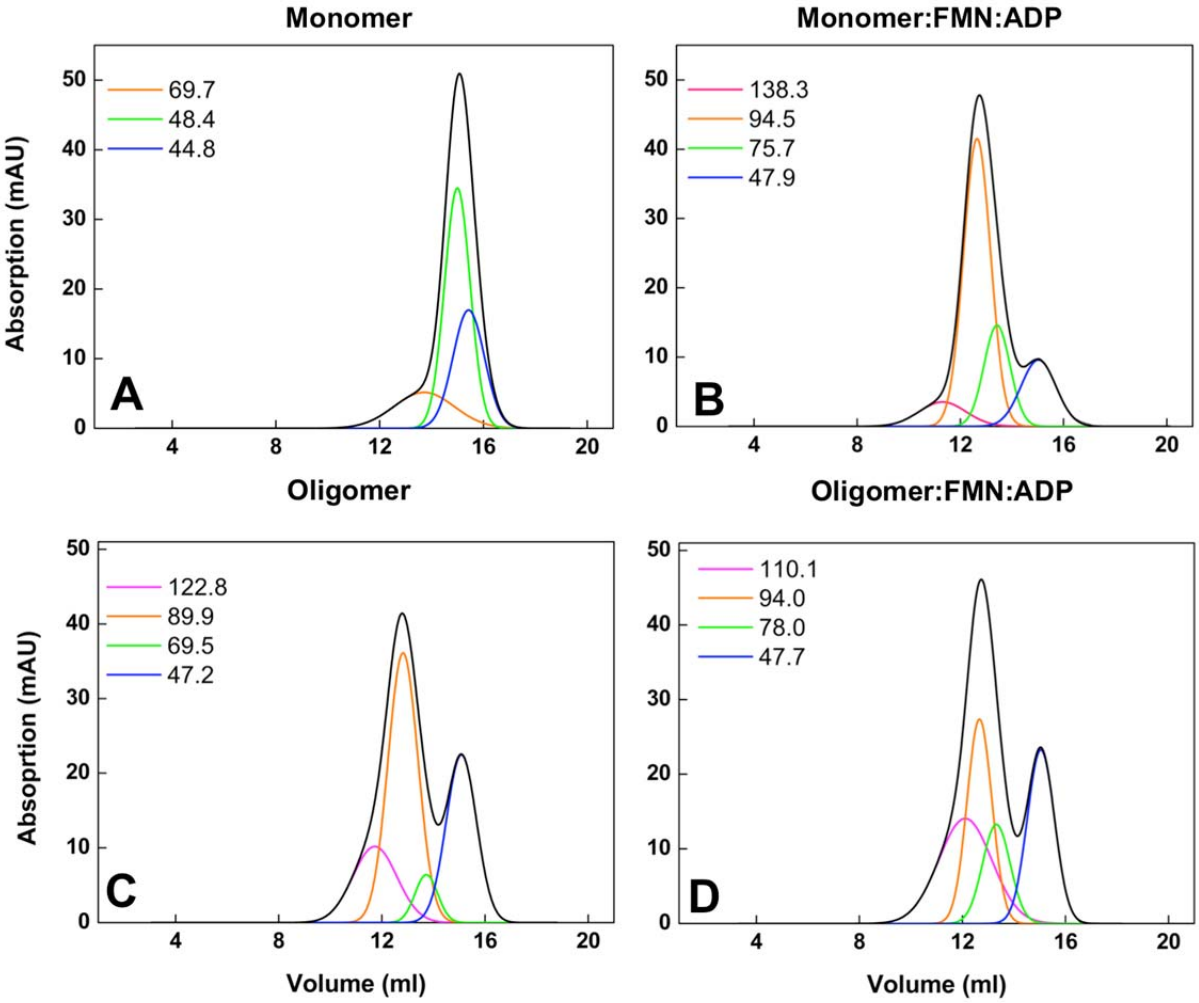

Figure SP8. Example of fitting analysis to a set of Gaussian functions of the gel filtration chromatograms obtained for E301K after incubation under the indicated conditions. The chromatograms are in black lines and the different populations assigned by the Gaussian analysis are in colored lines. The legend shows the calculated molecular weight for the peak maxima of every population.

Supporting tables

Table SP1. Thermodynamic parameters for the interaction of WT and mutant *Ca*FADSs with flavins and ATP determined by ITC. Data obtained at 25 °C in 20 mM PIPES, 10 mM $MgCl_2$, pH 7.0. Standard errors in Δ*G*, Δ*H* and –TΔ*S* where ±0.3 kcal/mol, taken in general larger than the standard deviation between replicates (n=3) and the numerical error after fitting analysis.

	**FADS:RF**			**FADS:FMN**			**FADS:FAD**			**FADS:ATP**		
	**Δ*G* (kcal/mol)**	**Δ*H* (kcal/mol)**	**-TΔ*S* (kcal/mol)**	**Δ*G* (kcal/mol)**	**Δ*H* (kcal/mol)**	**-TΔ*S* (kcal/mol)**	**Δ*G* (kcal/mol)**	**Δ*H* (kcal/mol)**	**-TΔ*S* (kcal/mol)**	**Δ*G* (kcal/mol)**	**Δ*H* (kcal/mol)**	**-TΔ*S* (kcal/mol)**
**WT**	-6.3	-7.5	1.2	-7.0	-35	28	-8.4	-26	18	-6.2	-30	23
**K202A**	-6.1	-4.2	-1.9	-6.7	-21.5	15	-7.1	-31	24	-5.7	-56	50
**E203A**	-6.8	-1.6	-5.2	-7.0	-13	6.2	-5.7	-41	35	-5.9	-24	18
**F206A**	n.d.[a]	n.d.[a]	n.d.[a]	-7.1	-6.7	-0.4	-7.8	-29	21	-6.6	-17	10.9
**F206K**	-7.0	-0.5	-6.5	-7.5	-12.5	5.0	-6.7	-17	10	-6.2	-24	17.4
**F206W**	-6.8	-9.9	3.1	-7.4	-19	11	-8.4	-18	9.1	-6.2	-23	17
**D298A**	-7.2	-1.5	-5.7	-6.4	-23	16	-7.5	-40	32	-6.1	-27	21
**D298E**	-6.5	-4.3	-2.2	-6.4	-23	16	-7.4	-56	48	-5.9	-38	32
**V300A**	-6.3	-11.2	4.9	-7.6	-10	2.8	-7.7	-13	5.7	-6.1	-30	24
**V300K**	n.d.[a]	n.d.[a]	n.d.[a]	n.d.[a]	n.d.[a]	n.d.[a]	-7.4	-5.9	-1.5	-6.6	-4.8	-1.8
**E301A**	-5.8	-13	6.7	-7.1	-13	6.1	-7.7	-13	5.8	-5.7	-39	33
**E301K**	-5.2	-59	54	-6.6	-22	15	-7.5	-24	16	-5.7	-48	42
**L304A**	-8.1	-5.9	-2.2	-6.5	-7.8	1.3	-6.2	-21	15	-6.3	-26	20
**L304K**	-6.0	-32.1	26.1	-6.0	-8.2	2.1	-6.3	-17	11	-6.0	-12	6.0

[a] No heat effects were detected in these ITC titrations.

Table SP2. Thermodynamic parameters for the interaction of FMN with the preformed WT and mutant *Ca*FADS:ADP complexes determined by ITC. Data obtained at 25 °C in 20 mM PIPES, 10 mM $MgCl_2$, pH 7.0. Standard errors in Δ*G*, Δ*H* and –TΔ*S* where ±0.3 kcal/mol, taken in general larger than the standard deviation between replicates (n=3) and the numerical error after fitting analysis.

	**FADS:ADP:FMN**					
	**Site 1**			**Site 2**		
	**Δ*G* (kcal/mol)**	**Δ*H* (kcal/mol)**	**-TΔ*S* (kcal/mol)**	**Δ*G* (kcal/mol)**	**Δ*H* (kcal/mol)**	**-TΔ*S* (kcal/mol)**
**WT**	-10	-4.1	-6.0	-8.2	-4.5	-3.8
**K202A**	-11	-3.3	-7.7	-7.8	-3.7	-4.3
**E203A**	-8.2	-1.4	-6.9	-6.9	-2.3	-4.5
**F206A**	-8.1	-6.3	-1.8	-6.8	-8.0	1.1
**F206K**	-8.7	-1.7	-7.1	-9.6	-2.3	-7.3
**F206W**	-9.9	-3.2	--6.7	-8.3	-1.6	-6.7
**D298A**	-8.1	-3.9	-4.2	-6.7	-8.9	2.2
**D298E**	-8.0	-1.7	-6.3	-7.5	-3.1	-4.3
**V300A**	-9.5	-15	5.6	-7.5	-15	7.9
**V300K**	-9.1	-5.5	-3.6	-7.7	-6.6	-1.1
**E301A**	-8.8	-3.0	-5.8	-7.3	-4.6	-2.7
**E301K**	-9.2	-2.7	-6.5	-6.7	-3.2	3.5
**L304A**	-8.6	-4.3	-4.3	-8.1	-4.9	-3.2
**L304K**	-7.9	-2.3	-5.6	-7.7	-6.3	-1.4

Table SP3. Predicted stability parameters for quaternary organizations inferred from the PISA server.

	Specie	Hexamer (A_3B_3)	Trimer B_3 (A_3)	Dimer (AB)
**Surface area**	WT	82800	45020 (44480)	31390
sq.·Å	F206W	83020	45070 (44530)[a]	30730
	D298E	81150	44540 (43380)[a]	30540
	E301A	84750	45690 (45660)[a]	31750
**Buried area**	WT	19770	6550 (6520)	2800
sq.·Å	F206W	17440	5840 (5020)[a]	2760
	D298E	18930	5960 (6210)[a]	2820
	E301A	18780	5830 (6340)[a]	2750
**ΔG_{int}** [b]	WT	-189.0	-73.0 (-72.0)	-52.7
kcal/mol	F206W	-200.4	-75.1 (-67.2)[a]	-51.9
	D298E	-181.3	-69.9 (-67.0)[a]	-48.3
	E301A	-175.4	-66.5 (-71.8)[a]	-45.0
**ΔG_{diss}** [c]	WT	22.5	3.1 (2.7)	12.4
kcal/mol	F206W	27.2	8.7 (-1.2)[a]	16.3
	D298E	19.6	2.2 (-1.4)[a]	12.3
	E301A	21.8	-0.5 (6.6)[a]	8.1

[a] These structures may or may not be stable in solution

[b] Indicates the solvation free energy gain upon formation of the assembly, in kcal/mol. The value is calculated as difference in total solvation energies of isolated and assembled structures. This value does not include the effect of satisfied hydrogen bonds and salt bridges across the assembly interfaces.

[c] Indicates the free energy of assembly dissociation, in kcal/mol. The free energy of dissociation corresponds to the free energy difference between dissociated and associated states. Positive values of ΔG^{diss} indicate that an external driving force should be applied in order to dissociate the assembly, therefore assemblies with $\Delta G^{diss} > 0$ are thermodynamically stable.

Table SP4. Data collection and refinement statistics for *Ca*FADS variants.

Crystal data	F206W	D298E	E301A
Space group	$P2_13$	$P2_13$	$P2_13$
Unit Cell Parameters (Å)	a = b = c = 133.48<br>a = b = γ = 90 °	a = b = c = 133.18<br>a = b = γ = 90 °	a = b = c = 134.96<br>a = b = γ = 90 °
**Data Collection**			
Temperature (K)	100	100	100
Beamline	ID14.1 ESRF	ID14.1 ESRF	ID14.1 ESRF
Wavelength (Å)	0.93340	0.93340	0.93340
Resolution (Å)	47.19 - 2.52 (2.65 -2.52)	47.09 - 2.51 (2.65 -2.51)	47.72 - 2.45 (2.58 -2.45)
Total reflections	238465 (21510)	287120 (26679)	257477 (20813)
Unique reflections	26874 (3670)	26982 (3788)	30328 (4208)
Mean I/σ(I)	24.2 (4.1)	24.7 (4.6)	19.0 (3.0)
Completeness (%)	99.1 (94.4)	99.6 (97.2)	92.2 (94.7)
Redundancy	8.9 (5.9)	10.6 (7.0)	8.5 (4.9)
R_{merge}[a]	0.078 (0.422)	0.085 (0.438)	0.080 (0.484)
**Data Refinement**			
Resolution range (Å)	40.0-2.52	45.0-2.51	40.0-2.45
Protein non-hydrogen atoms	5099	5142	5169
Ligand non-hydrogen atoms	28	28	28
Solvent non-hydrogen atoms	103	140	168
R_{work} (%)	20.96	20.33	20.90
R_{free}[b] (%)	26.28	25.08	25.61
r.m.s.d. bond length, (Å)	0.008	0.008	0.008
r.m.s.d. bond angles, (°)	1.252	1.216	1.160
Average B-factor, ($Å^2$)	35.71	35.84	38.37

Values in parentheses correspond to the highest resolution shell.
[a] $R_{merge} = \Sigma|I - I_{av}| / \Sigma I$, where the summation is over symmetry-equivalent reflection.
[b] R calculated for 7% of data excluded from the refinement.

REFERENCES

1 Frago, S., Velázquez-Campoy, A. & Medina, M. The puzzle of ligand binding to Corynebacterium ammoniagenes FAD synthetase. *J Biol Chem* **284**, 6610-6619, doi:M808142200 [pii] 10.1074/jbc.M808142200 (2009).

2 Serrano, A. *et al.* Key residues at the riboflavin kinase catalytic site of the bifunctional riboflavin kinase/FMN adenylyltransferase from Corynebacterium ammoniagenes. *Cell Biochem Biophys* **65**, 57-68, doi:10.1007/s12013-012-9403-9 (2013).

3 Herguedas, B., Martinez-Julvez, M., Frago, S., Medina, M. & Hermoso, J. A. Oligomeric state in the crystal structure of modular FAD synthetase provides insights into its sequential catalysis in prokaryotes. *J Mol Biol* **400**, 218-230, doi:S0022-2836(10)00497-3 [pii] 10.1016/j.jmb.2010.05.018 (2010).
